# Causal Effects of a Hepatic Senescence Gene Set on MASLD Fibrosis: A Mendelian Randomization Study and Quercetin Molecular Docking Analysis

**DOI:** 10.3390/biomedicines14030701

**Published:** 2026-03-17

**Authors:** Zhengwen Li, Yongzuo Li, Tianqing Jiang, Yue Wang, Zhengyou He

**Affiliations:** 1 School of Pharmacy, Chengdu University, 2025 Chengluo Avenue, Chengdu 610106, China; lizhengwen@cdu.edu.cn (Z.L.); 202216301104@cdu.edu.cn (Y.L.);; 2 Cell Biology-Inspired Tissue Engineering, Institute for Technology-Inspired, Regenerative Medicine, Maastricht University, 6200 MD Maastricht, The Netherlands; yue.wang@maastrichtuniversity.nl

**Keywords:** SHGS, fibrosis MASLD, Mendelian randomization, quercetin, molecular docking

## Abstract

**Background:** The senescence-associated hepatic gene set (SHGS) is critical in metabolic-dysfunction-associated steatotic liver disease (MASLD) progression. However, causal links between SHGS genes and liver diseases remain unclear. **Methods:** Mendelian randomization (MR) was used to explore causal relationships between SHGS genes and liver diseases. Immune infiltration of key genes was analyzed using the CIBERSORT algorithm with GEO database data, validated by single-cell RNA sequencing (scRNA-seq). Virtual docking assessed quercetin’s potential to modulate SHGS proteins and mitigate liver aging. **Results:** MR analysis identified elevated GBP2 expression as a risk factor for liver fibrosis (OR = 1.904, *p* = 0.028) but protective against cholangiocarcinoma (OR = 0.548, *p* = 0.001). Immune profiling and scRNA-seq revealed GBP2’s negative correlation with macrophages in fibrosis and positive correlations with T and NK cells in cholangiocarcinoma. Molecular docking suggested that quercetin indirectly suppresses GBP2 via IRF1, potentially attenuating liver aging. **Conclusions:** GBP2 might modulate hepatic fibrosis and cholangiocarcinoma. Quercetin may exert antifibrotic effects by indirectly modulating GBP2.

## 1. Introduction

Cellular senescence is an inevitable biological process primarily aimed at halting cellular replication in response to damage or replicative exhaustion [1]. Meanwhile, in chronic diseases, the persistent accumulation of senescent cells promotes pathogenesis [2]. Liver tissue, given its critical roles in drug metabolism and nutrient storage, is particularly susceptible to the adverse effects of cellular senescence [3]. Studies indicate that hepatic senescence manifests as progressive decline in hepatocyte function, impaired regeneration, and increased susceptibility to diseases such as MASLD, including non-alcoholic fatty liver, liver fibrosis, and even hepatocellular carcinoma (HCC) and hepatic cholangiocarcinoma (iCCA), which are associated with poor prognosis in liver disorders [4].

Metabolic alterations represent a hallmark of senescent hepatocytes, including dysregulated expression of glucose transporters GLUT2/4 and insulin-resistance-induced energy metabolism disruptions [5]. Research further demonstrates reduced activity of antioxidant enzymes (e.g., SOD, GSH-Px) in senescent cells, exacerbating oxidative damage [6]. Evidence shows pronounced activation of iron-dependent lipid peroxidation (ferroptosis) in aging hepatocytes, marked by upregulated ACSL4 expression [7]. Moreover, senescence exhibits functional heterogeneity across hepatic cell types: in parenchymal cells, it primarily impairs autophagy and promotes inflammation via SASP-mediated secretion of factors like IL-8. In senescent hepatic stellate cells (HSCs), TGF-β secretion activates fibroblasts, enhancing extracellular matrix deposition. Senescent macrophages, via IL-1β/IL-6 release, suppress immune surveillance, fostering a profibrotic microenvironment [3,8]. This functional heterogeneity among hepatic cell types complicates drug screening efforts.

Recent work by Du et al. [9] defined a senescence-associated hepatic gene set by identifying genes consistently upregulated in senescent hepatocytes across both in vitro and in vivo models. Using SHGS as a molecular marker, they subsequently identified senolytic compounds that selectively eliminate SHGS-positive hepatocytes, leading to improvements in MASLD in male mice. Although SHGS demonstrated robust performance across independent cohorts, it remains unclear whether enrichment of SHGS genes plays a causal role in MASLD progression or merely reflects a downstream consequence of liver disease. Given SHGS’s potential as a therapeutic target, elucidating its causal role holds significant translational value.

Mendelian randomization (MR) has emerged as a popular causal inference method utilizing genetic variants as instrumental variables [10]. It leverages the random allocation of alleles during meiosis to mimic randomized controlled trials (RCTs), thereby mitigating confounding and reverse causation biases inherent in observational studies. MR elucidates causal chains from “genetic variation → molecular phenotype → disease” and is widely applied in disease mechanism elucidation and drug target discovery [11,12]. Common instruments include expression quantitative trait loci (eQTLs), protein quantitative trait loci (pQTLs), methylation quantitative trait loci (meQTLs), or downstream biomarkers [13]. Considering SHGS as a gene set for hepatic senescence, we herein utilize eQTL datasets regulating gene expression as instrumental variables.

The current management of MASLD centers on lifestyle modification as the cornerstone, though long-term adherence remains challenging. Pharmacological approaches primarily target associated metabolic comorbidities. Drugs like GLP-1 receptor agonists and SGLT2 inhibitors show benefits for weight loss and glycemic control, with some evidence of histologic improvement [14]. Specific agents targeting disease progression, such as FXR agonists and PPAR agonists, are under investigation or recently approved, yet concerns regarding side effects, variable efficacy, and cost limit their universal application [15]. This unmet need has renewed interest in natural products. Herbal medicines have a long history in treating liver disorders, and modern research values their multi-target, multi-pathway actions—modulating inflammation, oxidative stress, metabolism, and fibrosis—which align well with the complex pathogenesis of MASLD [16]. This makes them promising sources for novel interventions. Among them, the flavonoid quercetin, widely present in fruits and vegetables, has emerged as a representative candidate due to its documented antioxidant, anti-inflammatory, and potential antifibrotic properties in preclinical studies, warranting further mechanistic exploration.

Quercetin, a flavonoid compound widely present in various fruits and traditional Chinese medicines such as Alpinia zerumbet, activates the nuclear factor erythroid 2-related factor 2 (Nrf2)/antioxidant response element (ARE) pathway [17,18,19]. This occurs via inhibition of Kelch-like ECH-associated protein 1 (Keap1), promoting Nrf2 nuclear translocation and upregulating antioxidant enzymes including superoxide dismutase (SOD) and glutathione peroxidase (GSH-Px), thereby enhancing hepatic antioxidant capacity. Additionally, quercetin activates the adenosine monophosphate-activated protein kinase (AMPK) pathway, suppressing fatty acid synthase (FAS) and acetyl-CoA carboxylase (ACC), while facilitating triglyceride catabolism to mitigate hepatic steatosis and insulin resistance [20]. Studies further indicate that improving quercetin’s bioavailability augments its antifibrotic efficacy [21]. These findings underscore quercetin’s pivotal role in attenuating liver aging. Accordingly, we will employ molecular dynamics simulations to investigate whether quercetin modulates proteins encoded by key senescence-associated hepatic gene set (SHGS) genes, laying the groundwork for subsequent anti-aging therapeutic development.

This study aims to employ Mendelian randomization to investigate the causal relationship between the senescence-related hepatic gene set (SHGS) and MASLD-related fibrosis, and to explore the potential therapeutic relevance of quercetin via bioinformatics and molecular docking analysis. The opposing roles of GBP2 in liver fibrosis and intrahepatic cholangiocarcinoma (iCCA) are reported as a secondary finding.

## 2. Materials and Methods

### 2.1. Data Source and Preprocessing

We utilized genome-wide association study (GWAS) summary statistics from seven liver disease datasets (https://gwas.mrcieu.ac.uk/, accessed on 1 June 2025), detailed in Table 1. The SHGS data, comprising 100 genes, was derived from Du et al. [9]. Corresponding genetic variants served as instrumental variables, with expression quantitative trait locus (eQTL) data sourced from the eQTLGen consortium (https://www.eqtlgen.org/cis-eqtls.html, accessed on 1 June 2025). To minimize population heterogeneity, all data were restricted to European ancestry.

### 2.2. Drug-Target Mendelian Randomization Analysis

Mendelian randomization (MR) analyses were conducted using the TwoSampleMR (v0.6.20) and MendelianRandomization R packages (Version: 0.10.0). Single-nucleotide polymorphisms (SNPs) were selected at *p* < 5 × 10^−8^, with linkage disequilibrium (LD) clumping performed at r^2^ = 0.3 and 100 kb window. Instrument strength was evaluated using R^2^ (variance in gene expression explained by SNPs) and F-statistics; higher F-values indicated stronger instruments, reducing weak instrument bias. Inverse-variance weighted (IVW) meta-analysis served as the primary method for causal inference [22], under the assumption of no horizontal pleiotropy. SNP effects were weighted to estimate the overall impact of gene expression on liver diseases. To detect and adjust for pleiotropy, MR-Egger regression was applied, with the Egger intercept used to assess directional bias; a non-zero intercept (*p* < 0.05) indicated potential pleiotropy [23]. Cochran’s Q test was used to assess heterogeneity among SNP effects.

To evaluate potential reverse causation, liver diseases were treated as exposures, with SNPs as instruments and gene expression as the outcome.

### 2.3. Bidirectional Mendelian Randomization

Bidirectional MR analyses followed the same procedures described above, with forward and reverse MR frameworks applied symmetrically to assess causal directionality between SHGS gene expression and liver disease outcomes.

### 2.4. Bioinformatics Analysis

The CIBERSORT [24] algorithm was employed to assess correlations between key SHGS gene expression and immune cell infiltration, with results visualized using ggplot2 [25]. Bulk RNA-seq data from GEO were analyzed (GSE135251: 206 MASLD cases, 10 controls, GSE179443: 59 iCCA cases). To delineate subcellular gene distribution, single-cell RNA-seq (scRNA-seq) data from GSE136103 (only CD45-, implied with 4 cirrhosis cases and 4 normal controls) and GSE138709 (5 iCCA cases, 3 adjacent controls) were processed using Seurat (v5) for normalization [26]; principal component analysis (PCA) and t-distributed stochastic neighbor embedding (t-SNE) enabled dimensionality reduction and clustering visualization, and cell subpopulations were annotated based on marker gene expression (Tables S3 and S4). Additionally, Gene Set Enrichment Analysis (GSEA) [27] analyses were performed on selected genes.

### 2.5. Molecular Docking

Upstream and downstream proteins of key targets were queried via STRING (https://string-db.org/, accessed on 1 June 2025). We used the default settings or a medium confidence threshold (>0.4) to retrieve functionally relevant interaction network centered on GBP2. Protein three-dimensional structures were retrieved from RCSB PDB (https://www.rcsb.org/, accessed on 1 June 2025). Quercetin structure was obtained from PubChem (https://pubchem.ncbi.nlm.nih.gov/, accessed on 1 June 2025) and optimized using the OPLS4 force field. For proteins lacking resolved structures, AlphaFold (v3) [28] predictions were utilized. Docking was performed with AutoDock (v4.2.6) [29]. During docking, we remove substances from protein structures, retaining only the target protein. Solvent water around protein was deleted beyond a distance of 5 Å. Missing hydrogens were set to fix when processing. We took the surface regions capable of forming stable interactions with molecules, such as hydrogen bonds or pi-pi conjugation, as binding sites, and generated about 420 results. These were ranked in ascending order of docking score, and the top three highest-scoring conformations corresponding to distinct, non-redundant binding modes were selected for evaluation and visualization.

## 3. Results

### 3.1. Causal Associations Between Variants in Multiple SHGS Genes and MASLD Development

**Figure 1:**
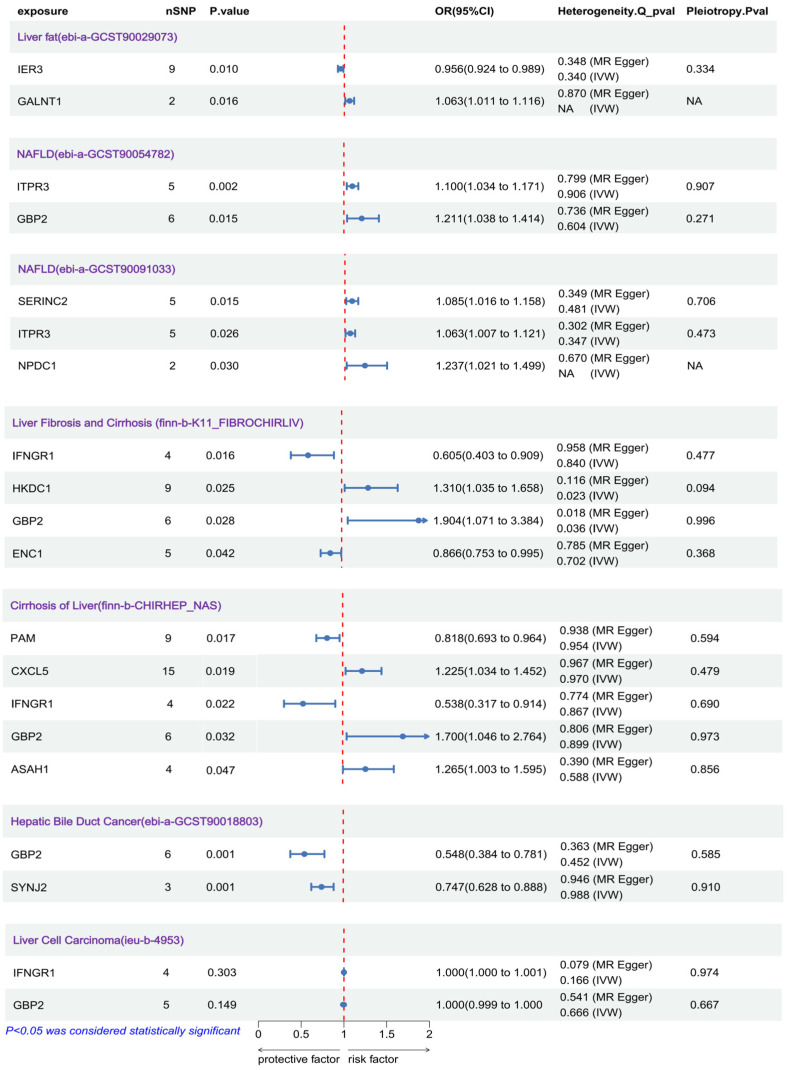
Mendelian randomization (MR) results showing causal relationships between SHGS genes and liver metabolism-related diseases (MR-Egger: Mendelian randomization–Egger; WM: weighted median; OR: odds ratio).

We analyzed causal relationships between genes in the senescence-associated hepatic gene set (SHGS) and multiple GWAS summary datasets, including fatty liver, MASLD, hepatic fibrosis, cirrhosis, hepatocellular carcinoma (HCC), and cholangiocarcinoma (hepatic bile duct cancer). Positive results are illustrated in the accompanying Figure 1.

In hepatic fibrosis and cirrhosis datasets, IFNGR1, HKDC1, GBP2, ENC1, and CD151 exhibited significant causal associations (*p* < 0.05 for all). Replication in a similar cohort (finn-b-CHIRHEP_NAS) confirmed the robustness of GBP2 as a risk factor for hepatic fibrosis (*p* = 0.032, OR = 1.70). Similarly, IFNGR1 showed a protective causal association against fibrosis (*p* = 0.022, OR = 0.538). GBP2 also demonstrated a causal effect in the MASLD cohort (*p* = 0.015, OR = 1.211). However, since MASLD includes both non-alcoholic fatty liver (NAFL) and metabolic-dysfunction-associated steatohepatitis (MASH), we could not distinguish whether this effect arose from the early NAFL stage or later NASH progression. The lack of standalone NAFL GWAS datasets precluded further confirmation. In intrahepatic cholangiocarcinoma (iCCA), higher GBP2 expression conferred a protective effect (*p* = 0.001, OR = 0.548), whereas no significant associations were observed in HCC datasets.

Heterogeneity analyses and horizontal pleiotropy tests yielded *p*-values > 0.05 for all positively associated genes, supporting the reliability of the causal estimates. Detailed graphical results, including scatter plots, funnel plots, and forest plots, are presented in Figures S1–S7. Proxy SNPs for each gene are listed in Table S1.

### 3.2. No Reverse Causality Between Key SHGS Gene Expression and MASLD

**Figure 2:**
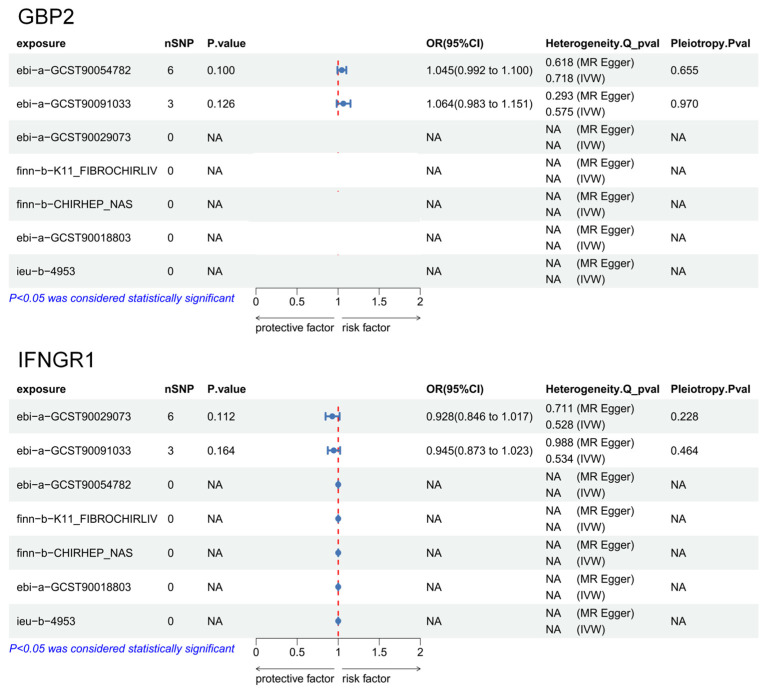
Reverse MR of GBP2 and IFNGR1 on liver metabolism-related diseases.

Given the clear causal links of GBP2 and IFNGR1 with hepatic fibrosis, we performed reverse MR analyses, treating liver diseases as exposures and SHGS genes as outcomes, to assess potential bidirectional effects. Results are shown in Figure 2.

Except for the MASLD and fatty liver datasets—where analyses showed sufficient valid SNPs but *p* > 0.05—no reverse causality was detected. Other analyses lacked valid SNPs, indicating no reverse causal effects of liver-related disease variants on IFNGR1 or GBP2 expression. Graphical results of reverse MR analyses are provided in Figures S8–S9, and associated SNPs are listed in Table S2.

### 3.3. GBP2 Is Related to Immune Infiltration in Hepatic Fibrosis and Cholangiocarcinoma with Divergent Trends

**Figure 3:**
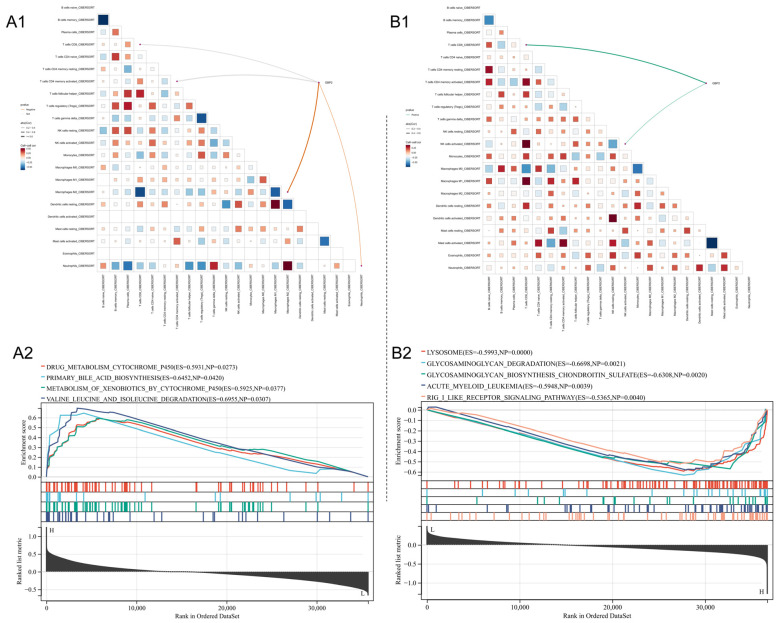
Immune infiltration analysis and GSEA results for GBP2. (**A1**): Immune infiltration in MASLD (GSE135251); orange line indicates a negative correlation. (**A2**): GSEA results based on MASLD samples. (**B1**): Immune infiltration in iCCA (GSE179443); green line indicates a positive correlation. (**B2**): GSEA results based on iCCA samples.

As interferon and inflammatory cytokines reportedly induce GBP2 expression, we assessed their relationship with immune cell infiltration in hepatic fibrosis and iCCA (Figure 3). For fibrosis analysis, only stage F3 and F4 samples from GSE135251 were used to enhance precision.

In hepatic fibrosis, GBP2 expression was negatively correlated with M2 macrophage infiltration (Figure 3(A1)). Conversely, in iCCA datasets (Figure 3(B1)), GBP2 showed a positive correlation with T-cell and NK-cell infiltration.

GSEA further revealed functional divergence. In fibrotic MASLD, GBP2 was associated with cytochrome P450-mediated drug metabolism and bile acid synthesis (Figure 3(A2)). In contrast, in iCCA, GBP2 was primarily linked to autophagy-related pathways, including lysosome function (Figure 3(B2)). This involvement in autophagy may underlie GBP2’s protective effect in iCCA.

### 3.4. Single-Cell Analysis of GBP2 Expression

**Figure 4:**
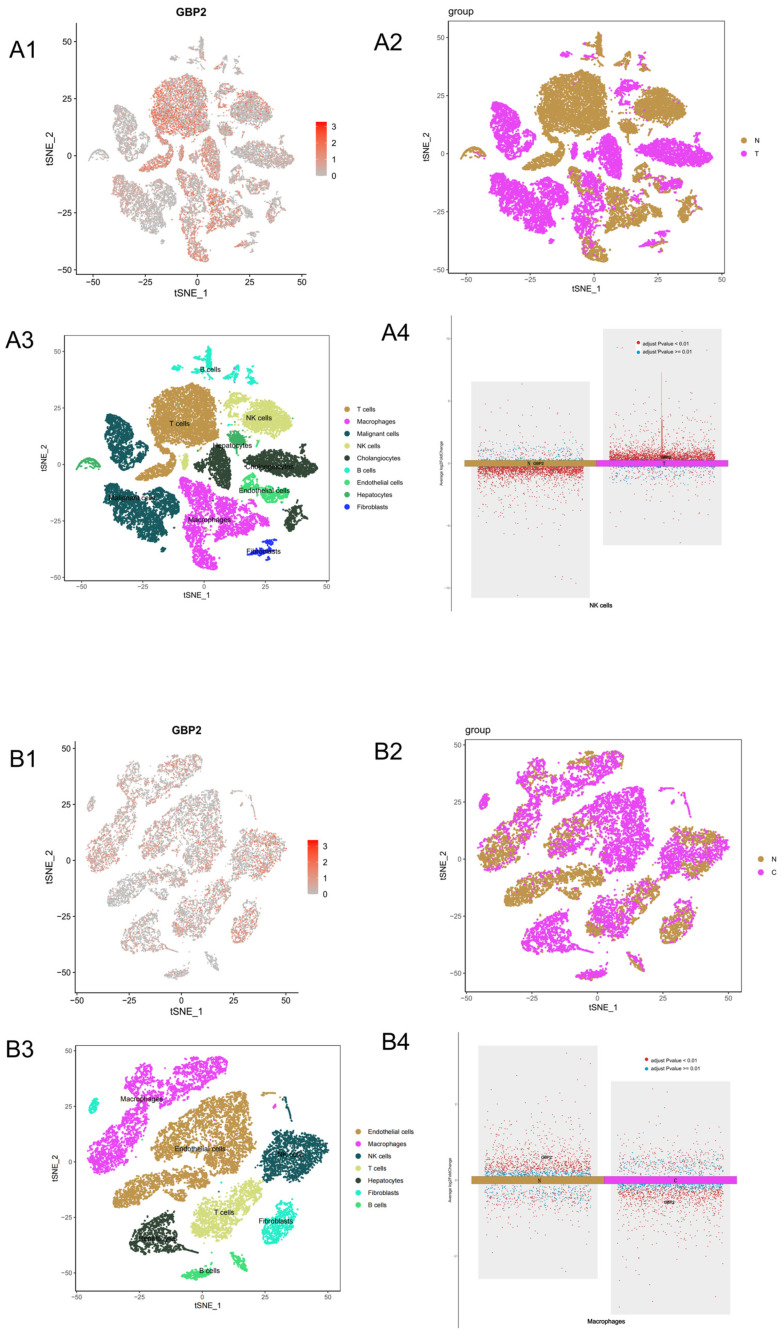
Single-cell RNA sequencing analysis from GSE138709 (iCCA) and GSE136103 (hepatic fibrosis). (**A1**) GBP2 expression across cell clusters of iCCA. (**A2**) Clustering by normal versus disease groups of iCCA. (**A3**) Cell clustering by type of iCCA. (**A4**) GBP2 expression in NK cells of iCCA; (**B1**) GBP2 expression across cell clusters of hepatic fibrosis. (**B2**) Clustering by normal versus disease groups of hepatic fibrosis. (**B3**) Cell clustering by type of hepatic fibrosis. (**B4**) GBP2 expression in macrophages of hepatic fibrosis; In (**B2**), “C” indicates cirrhosis cases.

Given GBP2’s impact on immune infiltration in liver diseases, we further analyzed its distribution across cell types in hepatic fibrosis and iCCA single-cell datasets (Figure 4).

In iCCA datasets, GBP2 showed broad distribution, with increased expression in NK cells compared to controls (Figure 4(A4)), consistent with bulk immune infiltration findings. T cells predominantly originated from normal samples in this dataset, precluding assessment of differential expression in T cells.

In hepatic fibrosis, GBP2 also exhibited widespread distribution. Its expression in macrophages was lower in fibrosis samples compared to controls (Figure 4(B4)), aligning with the negative correlation between GBP2 and macrophage infiltration observed in bulk RNA-seq analysis.

### 3.5. Molecular Docking Suggests Quercetin May Influence Hepatic Fibrosis Progression via IRF1

**Figure 5:**
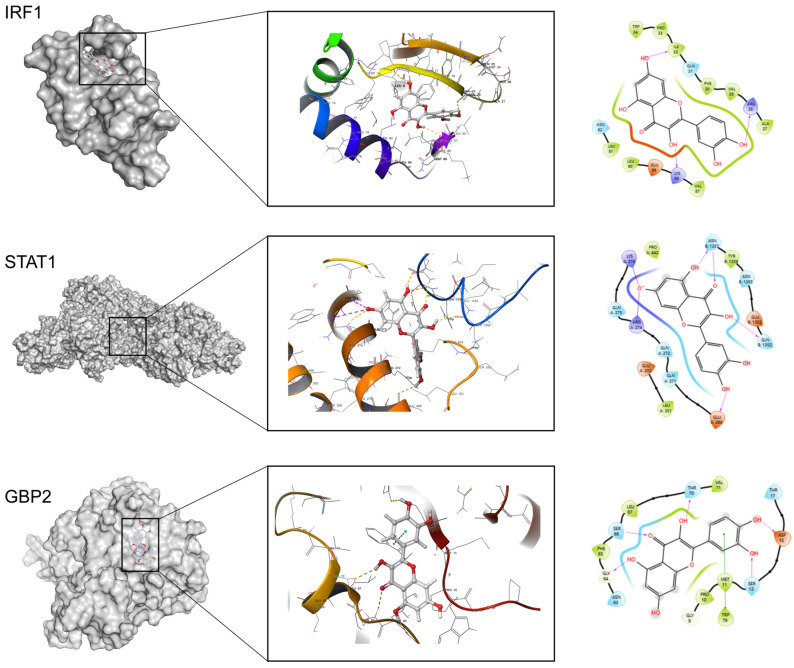
Docking result of quercetin with IRF1, STAT1 and GBP2 proteins.

Since flavonoids reportedly mitigate hepatic fibrosis, we performed molecular docking of quercetin—a representative flavonoid—with GBP2 to explore potential regulatory effects. Given that MR results might reflect upstream or downstream interactions, docking was also extended to GBP2-related proteins. Docking results are shown in Figure 5.

Docking identified 11 proteins with significant quercetin binding affinities. The top two targets were IRF1 (docking score: −8.805) and STAT1 (docking score: −8.792), whereas the docking score for GBP2 was [docking score: −6.135]. Additional docking results are presented in Figure S10. Quercetin exhibited hydrogen bond interactions with IRF1 residues ILE-32, ARG-28, and LYS-88, suggesting that quercetin may influence IRF1-related pathways, indirectly regulating GBP2 expression and thus modulating hepatic metabolism and aging.

## 4. Discussion

Mendelian randomization analysis revealed causal associations between genetic variants in multiple genes within SHGS and MASLD. Notably, variants in GBP2 exhibited significant roles across multiple disease stages, including hepatic fibrosis, cirrhosis, and iCCA, suggesting that GBP2 variants may contribute to disease progression.

GBP2, an interferon-inducible GTPase, exhibits constitutive expression in the liver, and has been hardly investigated in hepatic disease contexts. However, elevated GBP2 expression has been observed in MASLD patients [30]. Weighted gene co-expression network analysis in HCC demonstrated upregulated GBP2 at both RNA and protein levels, correlating with advanced tumor staging, increased patient age, and poor prognosis. This upregulation was also associated with alterations in the HCC immune microenvironment, including increased macrophage infiltration and reduced neutrophil and Th17 cell counts, indicating an immunomodulatory function of GBP2 in liver cancer [31]. However, our MR analysis found no causal link between GBP2 and HCC, implying that elevated GBP2 expression is a consequence rather than a cause of HCC. This was confirmed via reverse MR.

Intriguingly, while GBP2 overexpression conferred high risk in cirrhosis and fibrosis, it was associated with lower risk in iCCA. To elucidate this paradox, we examined GBP2’s relationship with immune infiltration. Analysis of GSE135251 data showed a negative correlation between GBP2 expression and macrophage infiltration in hepatic fibrosis, suggesting that GBP2 might suppress immune responses, thereby promoting fibrosis progression. Conversely, in iCCA, GBP2 was positively correlated with NK-cell infiltration, potentially underlying its protective effect. This aligns with our single-cell data observations, where GBP2 was upregulated in NK cells within cholangiocarcinoma tissues.

Another gene of interest is IFNGR1, encoding a key component of the interferon-γ (IFN-γ) receptor complex. We confirmed a causal association between IFNGR1 variants and hepatic fibrosis. IFNGR1 is essential for activating downstream JAK-STAT1 signaling, which regulates cell cycle arrest and inflammation in the liver. Complex crosstalk between IFN-γ signaling and pathways mediated by epidermal growth factor (EGF) and nuclear factor erythroid 2-related factor 2 (NRF2) can shift the hepatic microenvironment toward repair and regeneration [32,33]. In HBV-infected patients, IFN-γ therapy reduced liver progenitor cell proliferation and fibrosis, consistent with our MR findings that IFNGR1 variants conferring higher expression reduce fibrosis risk [34]. Notably, the upstream gene HKDC1 showed a causal association in the liver fibrosis and cirrhosis (finn-b-K11_FIBROCHIRLIV) cohort, with an odds ratio indicating increased fibrosis risk; however, this was not replicated in the cirrhosis of liver (finn-b-CHIRHEP_NAS) cohort, suggesting HKDC1’s role in fibrosis may be independent of IFNGR1 expression. No causal links were found between IFNGR1 variants and HCC or iCCA, possibly due to differing roles in the tumor microenvironment. STAT1 activation upregulates PD-L1, an immunosuppressive molecule that inhibits CD8+ T-cell activity, facilitating HCC immune escape and tumor growth.

Quercetin, a flavonoid, has been shown to delay hepatic aging, though its mechanisms remain unclear. We investigated via virtual docking whether quercetin influences hepatic aging through GBP2-related proteins. Results indicated indirect effects via upstream proteins IRF1 (docking score: −8.805), rather than direct binding to GBP2. This concurs with prior studies showing quercetin’s inhibition of IRF1 activation [35]. As an upstream transcription factor, IRF1 participates in interferon signaling and immune responses, binding to the GBP2 promoter to enhance its transcription. In fibrosis, where elevated GBP2 is a risk factor, quercetin’s indirect modulation of GBP2 via IRF1 may contribute to delaying hepatic aging.

Although GBP2 is biologically relevant in the context of MASLD, the MR analysis in this study is based on mixed etiological fibrosis data; it should be noted that corroboration using imaging, histologic fibrosis scoring, or biochemical markers in independent cohorts would strengthen the selection of GBP2 as a key gene for our analysis. Therefore, the conclusion that GBP2 was identified as a risk factor for liver fibrosis should be understood to apply to the broader progression of liver fibrosis, not just MASLD-driven fibrosis. In addition, the size of the dataset itself may also cause the results to be biased, especially for the scRNA-seq analysis of GSE136103, which is underpowered for formal statistical inference; although batch effect correction was performed using Harmony prior to clustering and visualization, it should be noted that these single-cell observations should be regarded as hypothesis-generating and require replication in larger cohorts. Establishing the specific causal role of GBP2 in MASLD fibrosis requires validation using large-scale GWAS data on MASLD or MASH-related fibrosis with clearly defined etiologies.

It should also be noted that, beyond inherent MR limitations, such as potential horizontal pleiotropy, the reliability of our findings depends on the quality of the GWAS summary datasets. Additionally, as eQTL data were blood-derived, potential tissue-specific expression biases may limit generalizability. Finally, absence of causal associations for other SHGS genes does not exclude their biological relevance in hepatic diseases.

## 5. Conclusions

By integrating Mendelian randomization and bio-informatics analysis, this study suggests a potential causal link between liver-aging-related genes and liver diseases. Specifically, GBP2 was identified as a key gene through MR analysis, suggesting a potential causal role in liver fibrosis and intrahepatic cholangiocarcinoma, possibly mediated through the regulation of immune cell infiltration. Furthermore, molecular docking and literature analysis indicate that the flavonoid quercetin may indirectly regulate GBP2 through the upstream transcription factor IRF1, thereby potentially influencing the fibrotic process. These findings highlight GBP2 as a promising candidate therapeutic target and provide experimentally verifiable hypotheses for further exploration of the quercetin-IRF1-GBP2 axis in liver aging and fibrosis. However, this regulatory pathway is currently still a hypothesis based on bioinformatics predictions and literature deductions. Its validity needs to be directly verified through subsequent cell experiments (such as detecting IRF1 activity, GBP2 expression, and downstream phenotypes after quercetin treatment) and animal models.

## Figures and Tables

**Table 1:** Characteristics of the GWAS cohorts.

Characteristics	GWAS-ID	Type	Sample Size	Number of SNPs
Liver fat	ebi-a-GCST90029073	Outcome	32,974	9,499,333
NAFLD/MASLD	ebi-a-GCST90054782	Outcome	377,988	9,097,254
NAFLD/MASLD	ebi-a-GCST90091033	Outcome	778,164	6,784,388
Liver fibrosis and firrhosis	finn-b-K11_FIBROCHIRLIV	Outcome	214,403	16,380,458
Cirrhosis of liver	finn-b-CHIRHEP_NAS	Outcome	217,334	16,380,465
Hepatic bile duct cancer	ebi-a-GCST90018803	Outcome	476,091	24,196,592
Liver cell carcinoma	ieu-b-4953	Outcome	372,184	6,304,034

## Data Availability

All data used in this study are available in a public repository. The code involved in the data analysis process can be obtained by contacting the corresponding author.

## Supplementary Materials

The following supporting information can be downloaded at https://www.mdpi.com/article/10.3390/biomedicines14030701/s1. Table S1: MR: Final IVs of SHGS for liver diseases; Table S2: Reverse MR of GBP2 and IFNGR1 on liver metabolism-related diseases; Figure S1: MR Graphical results of Liver fat; Figure S2. MR Graphical results of MASLD(ebi-a-GCST90054782); Figure S3. MR Graphical results of MASLD(ebi-a-GCST90091033); Figure S4. MR Graphical results of Liver Fibrosis and cirrhosis(finn-b-K11_FIBROCHIRLIV); Figure S5. MR Graphical results of cirrhosis of liver (finn-b-CHIRHEP_NAS); Figure S6. MR Graphical results of Hepatic Bile Duct Cancer (ebi-a-GCST90018803); Figure S7. MR Graphical results of Liver cell carcinoma (ieu-b-4953); Figure S8. Reverse MR of GBP2 on MASLD(ebi-a-GCST90054782) (UP)and iCCA(ebi-a-GCST90018803)(DOWN); Figure S9. Reverse MR of IFNGR1 on iCCA(ebi-a-GCST90018803) (UP)and liver fat(ebi-a-GCST90029073)(DOWN); Figure S10. Docking results of quercetin with GBP2 related proteins; Table S3: Cell-type-specific genes for GSE138709; Table S4: Cell-type-specific genes for GSE136103.
