# Supplementary material for: Causal Effects of a Hepatic Senescence Gene Set on MASLD Fibrosis: A Mendelian Randomization Study and Quercetin Molecular Docking Analysis
Source: Biomedicines. 2026 Mar 17;14(3):701. doi: 10.3390/biomedicines14030701 (PMC13024729; doi:10.3390/biomedicines14030701)
Supplement: Supplementary file 1 [file biomedicines-14-00701-s001.zip › biomedicines-4160329-supplementary.pdf]

Supplementary:

Causal Effects of a Hepatic Senescence Gene Set on MASLD Fibrosis: A Mendelian Randomization Study and Quercetin Molecular Docking Analys

Table.S1 MR: Final IVs of SHGS for Liver Diseases

Ivs	Chr	EA	ALT	β	SE	p1	p2	Gene name	
rs149007767	7	T	C	0.126555	0.0166476	2.92012e-14	0.0469105	GBP2	iCCA(ebi-a-GCST90018803)
rs2182322	1	G	A	0.233605	0.0337219	4.28647e-12	0.1328		
rs4788084	16	T	C	-0.0862661	0.0120384	7.72147e-13	0.0541003		
rs653178	12	T	C	-0.177081	0.0118484	1.6638e-50	0.0272001		
rs76830965	3	A	C	0.120281	0.018856	1.7848e-10	0.5322		
rs9264638	6	A	G	0.0916847	0.0127059	5.35427e-13	0.4525		
rs17489570	6	A	G	0.846512	0.0184715	1e-200	0.00185102	SYNJ2	
rs661857	6	C	T	-0.143809	0.0118431	6.24885e-34	0.3649		
rs827956	6	A	G	0.100681	0.0122074	1.61883e-16	0.5463		
rs149007767	7	T	C	0.126555	0.0166476	2.92012e-14	0.780001	GBP2	Liver cell carcinoma(ieu-b-4953)
rs4788084	16	T	C	-0.0862661	0.0120384	7.72147e-13	0.52		
rs653178	12	T	C	-0.177081	0.0118484	1.6638e-50	0.0719996		
rs76830965	3	A	C	0.120281	0.018856	1.7848e-10	0.54		
rs9264638	6	A	G	0.0916847	0.0127059	5.35427e-13	0.55		
rs11754268	6	T	C	-0.241928	0.0138938	6.62064e-68	0.12	IFNGR1	
rs2594836	5	A	G	0.113683	0.0137052	1.08593e-16	0.2		
rs56388170	7	T	G	0.0756882	0.0131119	7.81502e-09	0.649999		
rs7210990	17	A	C	0.0694576	0.0118969	5.27679e-09	0.11		
rs149007767	7	T	C	0.126555	0.0166476	2.92012e-14	0.781927	GBP2	NAFLD(ebi-a-GCST90054782)
rs2182322	1	G	A	0.233605	0.0337219	4.28647e-12	0.894073		
rs4788084	16	T	C	-0.0862661	0.0120384	7.72147e-13	0.293371		
rs653178	12	T	C	-0.177081	0.0118484	1.6638e-50	0.167406		
rs76830965	3	A	C	0.120281	0.018856	1.7848e-10	0.387663		
rs9264638	6	A	G	0.0916847	0.0127059	5.35427e-13	0.0132309		
rs114746521	6	A	G	0.350115	0.047085	1.04088e-13	0.735028	ITPR3	
rs210137	6	T	C	-0.595795	0.0142423	1e-200	0.122484		
rs58649023	6	G	A	-0.309788	0.0264067	8.77809e-32	0.256919		
rs6915136	6	C	T	0.565635	0.0160559	1e-200	0.0197156		
rs73410764	6	T	C	-0.17105	0.0267136	1.52149e-10	0.728471		
rs1128175	6	G	A	0.117956	0.0137674	1.05487e-17	0.39		
rs114071505	6	C	G	-0.162073	0.0289245	2.10189e-08	0.7		

rs114708313	6	T	A	-0.144876	0.0214453	1.42266e-11	0.91	IER3	liver fat(ebi-a-GCST 90029073)
rs1264372	6	T	C	0.43847	0.0134623	1e-200	0.0329997		
rs149110519	6	T	C	-0.290323	0.0265455	7.67008e-28	0.00739997		
rs2233966	6	G	A	0.169879	0.0118212	7.91407e-47	0.0790005		
rs56330463	5	C	T	-0.0751493	0.0120507	4.489e-10	0.51		
rs7210990	17	A	C	0.078799	0.0118928	3.4586e-11	0.58		
rs77526211	13	A	G	-0.197352	0.029224	1.44611e-11	0.94		
rs4799373	18	G	A	-0.0868679	0.0118922	2.78099e-13	0.64	GALNT1	
rs522964	18	G	A	-0.295007	0.0116428	1.20504e-141	0.012		
rs116679227	1	T	C	0.287766	0.0432296	2.80221e-11	0.6138	SERINC2	NAFLD(eqtl-a-ENSG00000107281)
rs12139418	1	C	T	0.457802	0.0114057	1e-200	0.0399797		
rs12480732	20	T	C	-0.0808818	0.0136585	3.18442e-09	0.8937		
rs143751725	1	T	C	-0.234434	0.0381057	7.63185e-10	0.779301		
rs71644192	1	T	C	-0.346024	0.0301667	1.85353e-30	0.0275398		
rs114746521	6	A	G	0.350115	0.047085	1.04088e-13	0.760599	ITPR3	
rs210137	6	T	C	-0.595795	0.0142423	1e-200	0.1238		
rs58649023	6	G	A	-0.309788	0.0264067	8.77809e-32	0.1256		
rs6915136	6	C	T	0.565635	0.0160559	1e-200	0.0806102		
rs73410764	6	T	C	-0.17105	0.0267136	1.52149e-10	0.1418		
rs2784074	9	T	C	0.175748	0.0143561	1.84969e-34	0.0391003	NPDC1	
rs55908509	19	A	G	-0.0955772	0.0126477	4.12477e-14	0.4177		
rs149007767	7	T	C	0.126555	0.0166476	2.92012e-14	0.8143	GBP2	Fibrosis and chirrhosis of liver(finn-b-K11_FIBROCHIRLIV)
rs4788084	16	T	C	-0.0862661	0.0120384	7.72147e-13	0.646501		
rs653178	12	T	C	-0.177081	0.0118484	1.6638e-50	0.04509		
rs76830965	3	A	C	0.120281	0.018856	1.7848e-10	0.0001456		
rs9264638	6	A	G	0.0916847	0.0127059	5.35427e-13	0.1974		
rs11754268	6	T	C	-0.241928	0.0138938	6.62064e-68	0.1288	IFNGR1	
rs2594836	5	A	G	0.113683	0.0137052	1.08593e-16	0.239		
rs56388170	7	T	G	0.0756882	0.0131119	7.81502e-09	0.1729		
rs7210990	17	A	C	0.0694576	0.0118969	5.27679e-09	0.284		
rs10998645	10	C	T	-0.542256	0.0115722	1e-200	0.0146501	HKDC1	
rs11755527	6	G	C	0.0782376	0.0119892	6.7733e-11	0.1046		
rs117985078	10	C	T	0.377906	0.0454111	8.66363e-17	0.768		
rs118090407	10	T	C	-0.681729	0.0275826	7.19449e-135	0.4316		
rs13108043	4	A	G	0.110755	0.0158337	2.65644e-12	0.4933		
rs3184504	12	C	T	-0.0656163	0.0119331	3.82384e-08	6.05703e-05		
rs55908509	19	A	G	-0.121869	0.0126317	5.01187e-22	0.6497		
rs7094214	10	A	G	-0.107863	0.0157498	7.45762e-12	0.8409		
rs77380489	10	A	G	0.255122	0.0291309	1.99434e-18	0.0393804		
rs9790517	4	T	C	-0.0803621	0.0139401	8.17542e-09	0.2891		
rs9790517	4	T	C	-0.0803621	0.0139401	8.17542e-09	0.2891		
rs149425066	5	A	G	-0.227539	0.0366295	5.23142e-10	0.4536	ENC1	

rs35288236	5	T	C	0.109363	0.018111	1.5565e-09	0.4043		
rs412467	5	G	A	0.140037	0.0140373	1.93731e-23	0.3689		
rs79905307	5	T	C	0.560984	0.0399203	7.42506e-45	0.1571		
rs9293628	5	A	C	1.29077	0.0171285	1e-200	0.133		
rs149007767	7	T	C	0.126555	0.0166476	2.92012e-14	0.1956	GBP2	Chirrosis of liver(finn-b-CHIRHEP_NAS)
rs2182322	1	G	A	0.233605	0.0337219	4.28647e-12	0.9784		
rs4788084	16	T	C	-0.0862661	0.0120384	7.72147e-13	0.5023		
rs653178	12	T	C	-0.177081	0.0118484	1.6638e-50	0.0455596		
rs76830965	3	A	C	0.120281	0.018856	1.7848e-10	0.9702		
rs9264638	6	A	G	0.0916847	0.0127059	5.35427e-13	0.7858		
rs11754268	6	T	C	-0.241928	0.0138938	6.62064e-68	0.02266	IFNGR1	
rs2594836	5	A	G	0.113683	0.0137052	1.08593e-16	0.8245		
rs56388170	7	T	G	0.0756882	0.0131119	7.81502e-09	0.6012		
rs7210990	17	A	C	0.0694576	0.0118969	5.27679e-09	0.4997		
rs1050316	1	T	G	-0.118175	0.0125487	4.63234e-21	0.9571	CXCL5	
rs10512472	17	C	T	0.14659	0.0154752	2.73464e-21	0.4736		
rs10821556	9	A	C	-0.108894	0.0125059	3.10384e-18	0.1745		
rs114199908	4	G	A	0.766575	0.0479535	1.60472e-57	0.1413		
rs114694170	5	C	T	0.153094	0.0266983	9.79738e-09	0.6462		
rs11602954	11	A	G	0.0923724	0.0145471	2.15601e-10	0.9235		
rs12447718	16	A	G	-0.153548	0.0276433	2.77933e-08	0.4268		
rs1354034	3	C	T	0.249896	0.0120684	3.00815e-95	0.567599		
rs329121	5	C	G	-0.0673323	0.0120782	2.48102e-08	0.3759		
rs34426467	2	A	T	0.0713182	0.0121849	4.82203e-09	0.751		
rs35304300	6	G	A	-0.0824044	0.0144238	1.10869e-08	0.8066		
rs56078309	4	A	G	1.00981	0.0204055	1e-200	0.3001		
rs6993770	8	T	A	0.210001	0.0132281	9.3821e-57	0.6996		
rs7075195	10	G	A	0.249869	0.011869	2.1923e-98	0.1097		
rs79755767	12	G	C	0.11095	0.0200206	2.99723e-08	0.3356		
rs10038686	5	A	G	-0.205678	0.0127167	7.69308e-59	0.8295	PAM	
rs141290553	5	T	G	0.53751	0.0520656	5.51188e-25	0.8009		
rs17375145	5	G	A	-0.223	0.0232594	9.01571e-22	0.852		
rs2033562	8	C	G	0.0676369	0.0122688	3.52769e-08	0.736999		
rs438617	5	C	T	-0.728576	0.012128	1e-200	0.03322		
rs4455005	17	A	G	-0.0699627	0.0122372	1.0829e-08	0.8594		
rs56314246	5	A	G	0.638979	0.0180829	1e-200	0.3095		
rs6866114	5	T	A	-0.263251	0.0347063	3.31971e-14	0.3995		
rs77526211	13	A	G	-0.164101	0.0292385	1.9931e-08	0.1781		
rs10503644	8	G	A	-0.473832	0.0112675	1e-200	0.1035	ASAH1	
rs117352317	8	G	A	0.473247	0.0453532	1.72346e-25	0.2777		
rs11785618	8	T	C	-0.150237	0.0228604	4.96478e-11	0.8445		
rs4087059	8	G	T	-0.342898	0.0145315	4.15911e-123	0.1555		

Chr:chromosome;EA:effect allele;ALT: alternative allele(Other allele):p1：Association of SNPs with exposures；p2:Association of individual SNPs with outcomes

Table.S2 Reverse MR of GBP2 and IFNGR1 on liver metabolism related diseases

Ivs	Chr	EA	ALT	β	SE	p1	p2	Gene name	
rs1260326	2	C	T	-0.136025	0.020866	2.5363e-11	0.73687	GBP2	NAFLD(ebi-a-GCST90054782)
rs17321515	8	G	A	-0.154093	0.0207828	1.81343e-13	0.156834		
rs2642442	1	T	C	0.13769	0.0227583	7.67132e-10	0.867527		
rs3747207	22	A	G	0.369714	0.0229366	6.74062e-60	0.0743909		
rs429358	19	C	T	-0.199223	0.0304411	2.1692e-11	0.637354		
rs73001065	19	C	G	0.345021	0.0348771	1.08143e-24	0.844141		
rs3747207	22	A	G	0.288601	0.019822	5.07341e-48	0.0743909	GBP2	iCCA(ebi-a-GCST90018803)
rs429358	19	C	T	-0.136615	0.0239288	1.13501e-08	0.637354		
rs73001065	19	C	G	0.281009	0.0326406	7.35699e-18	0.844141		
rs3747207	22	A	G	0.288601	0.019822	5.07341e-48	0.138021	IFNGR1	iCCA(ebi-a-GCST90018803)
rs429358	19	C	T	-0.136615	0.0239288	1.13501e-08	0.53618		
rs73001065	19	C	G	0.281009	0.0326406	7.35699e-18	0.43351		
rs112875651	8	A	G	-0.0500699	0.00799546	3.79997e-10	0.465146	IFNGR1	liver fat(ebi-a-GCST90029073)
rs1229984	4	C	T	0.157856	0.0253448	7.00003e-10	0.152717		
rs2250802	10	A	G	-0.0539399	0.00867948	1.40001e-09	0.74609		
rs429358	19	C	T	-0.12145	0.010762	1.50003e-29	0.53618		
rs58542926	19	T	C	0.289217	0.0147025	2.80027e-85	0.233575		
rs738408	22	T	C	0.194685	0.00941686	5.30029e-95	0.138021		

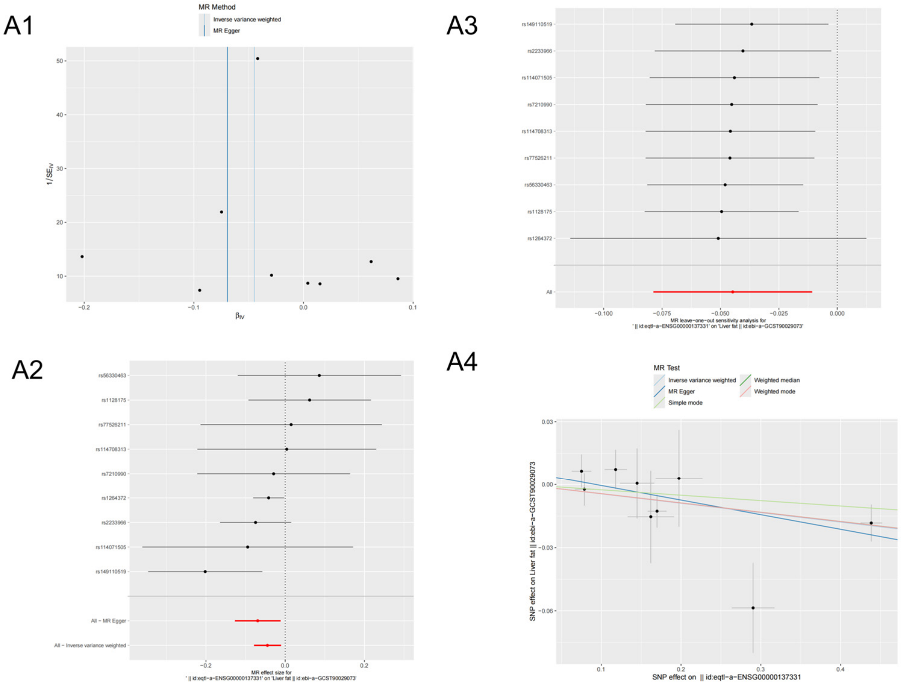

IER3

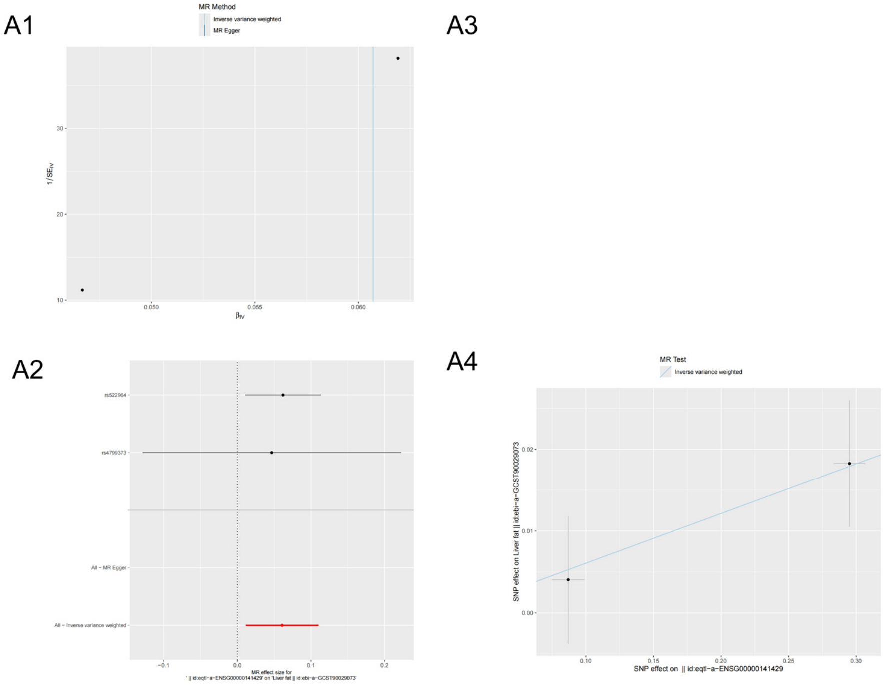

GALNT1

Figure S1. MR Graphical results of Liver fat .A1: funnel plots ; A2: OR scatter plots; A3:Leave-One-Out plots; A4: OR forest plots. Due to lack of SNPs, leave-one-out polt might

not be pictured.OR=Odds Ratio

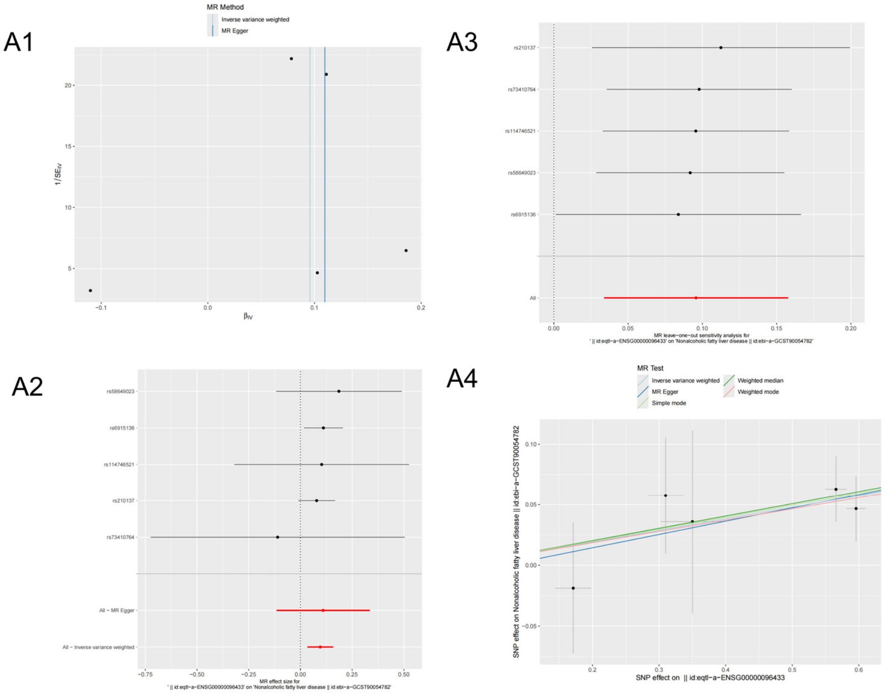

ITPR3

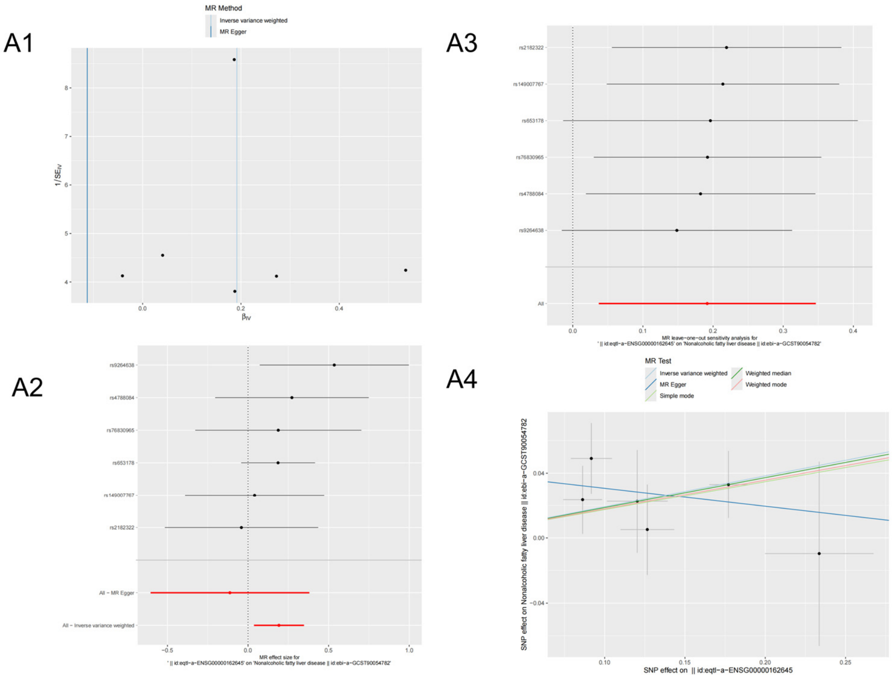

GBP2

Figure S2. MR Graphical results of MASLD(ebi-a-GCST90054782) .A1: funnel plots ; A2: OR scatter plots; A3:Leave-One-Out plots; A4: OR forest plots.OR=Odds Ratio

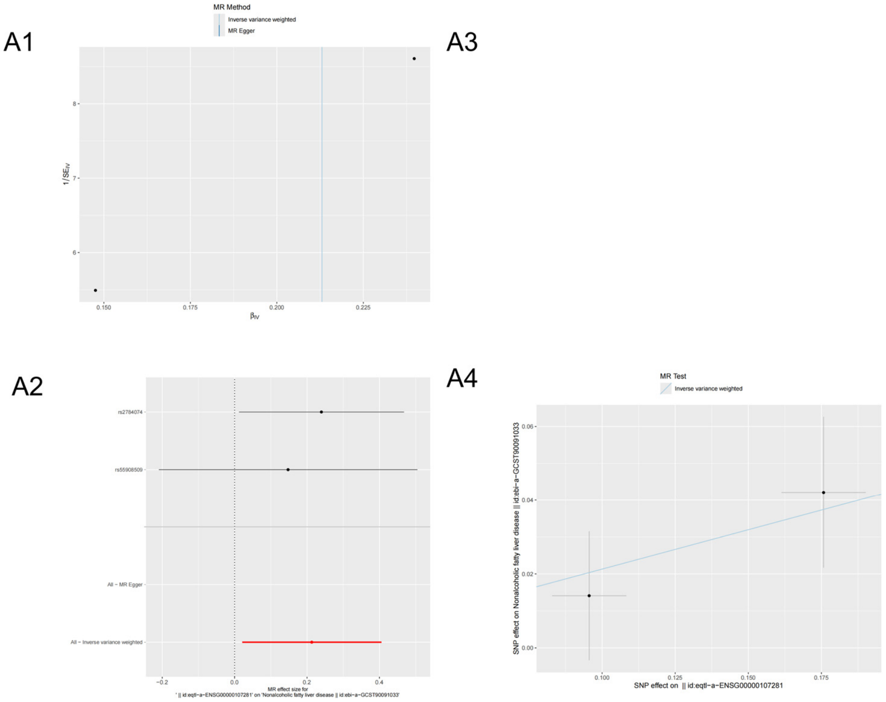

NPDC1

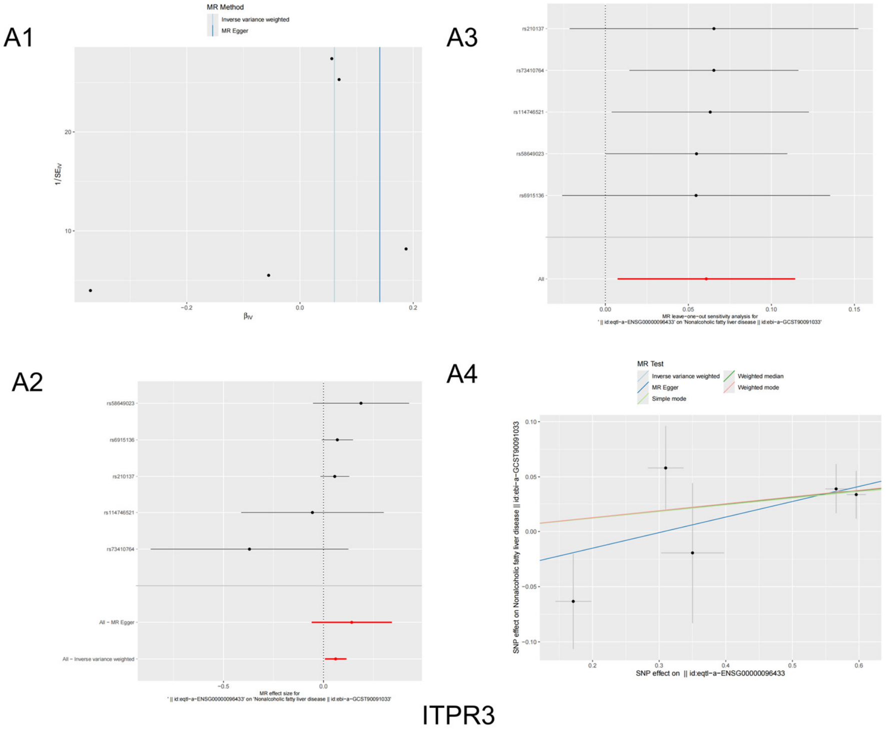

ITPR3

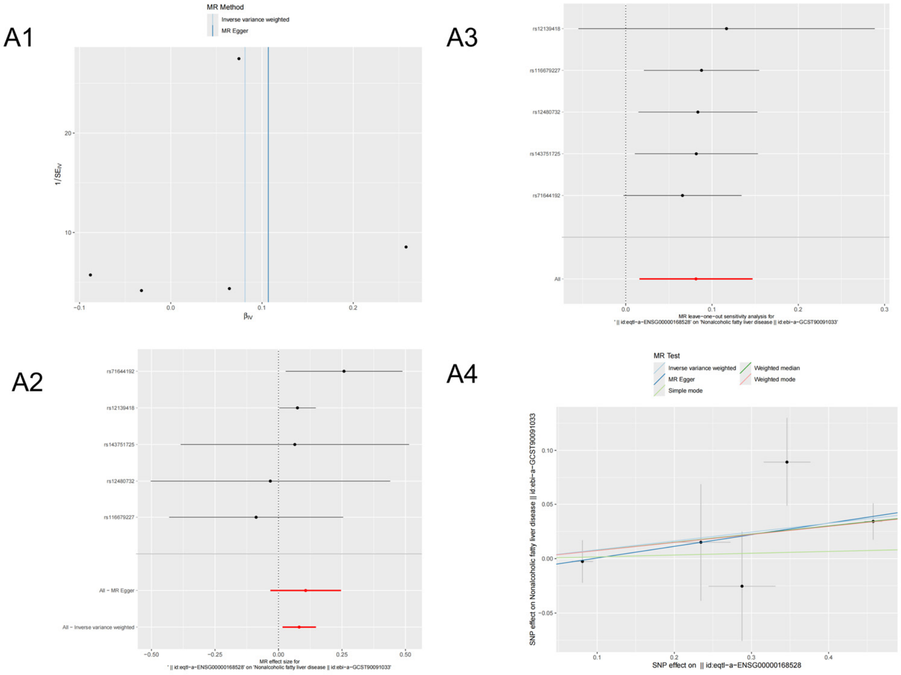

SERINC2

Figure S3. MR Graphical results of MASLD(ebi-a-GCST90091033) .A1: funnel plots ; A2: OR scatter plots; A3:Leave-One-Out plots; A4: OR forest plots. Due to lack of SNPs, leave-one-out polt might not be pictured.OR=Odds Ratio

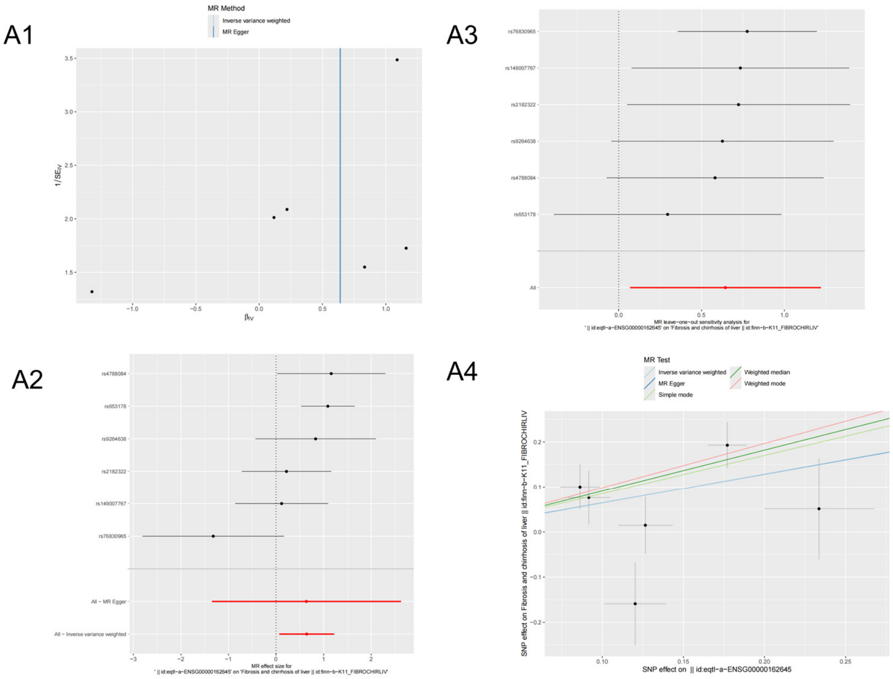
A1
A3
A2
A4
MR Method
Inverse variance weighted
MR Egger
MR Test
Inverse variance weighted
MR Egger
Simple mode
Weighted median
Weighted mode
SNP effect on || id:eqtl-a-ENSG00000162645

GBP2

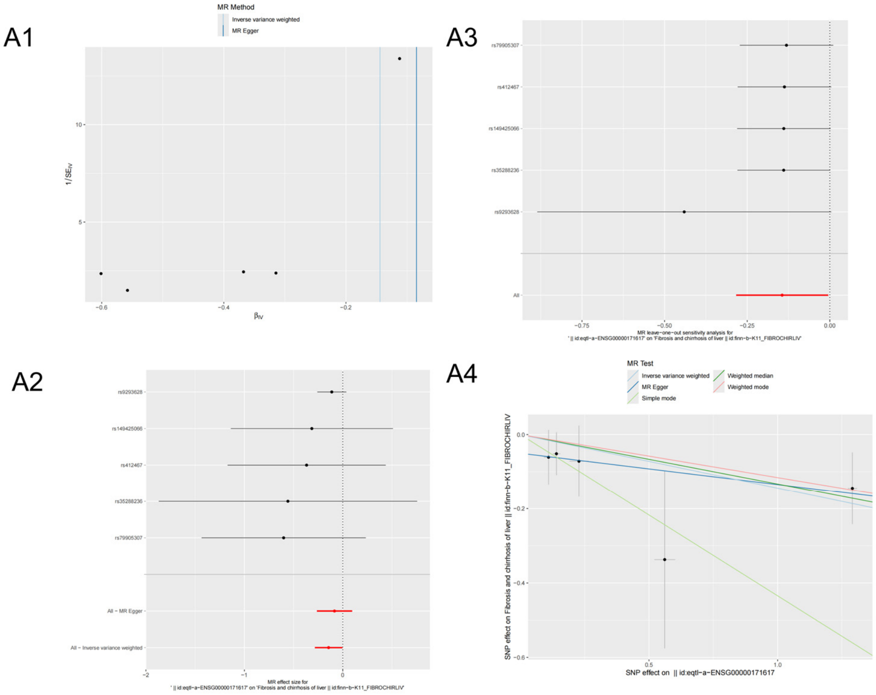
A1
A3
A2
A4
MR Method
Inverse variance weighted
MR Egger
MR Test
Inverse variance weighted
MR Egger
Simple mode
Weighted median
Weighted mode
SNP effect on || id:eqtl-a-ENSG00000171617

ENC1

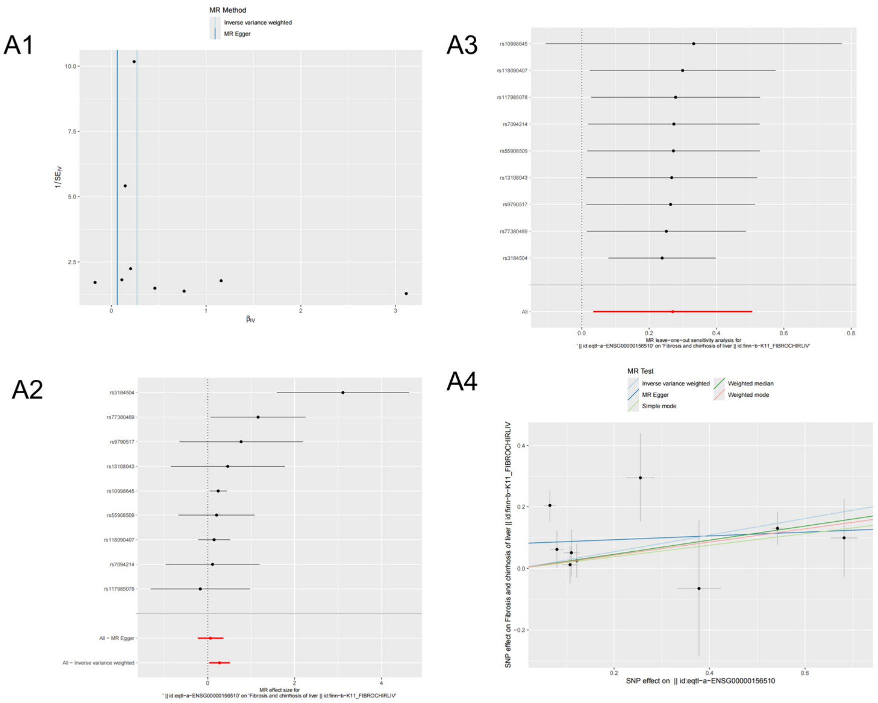

HKDC1

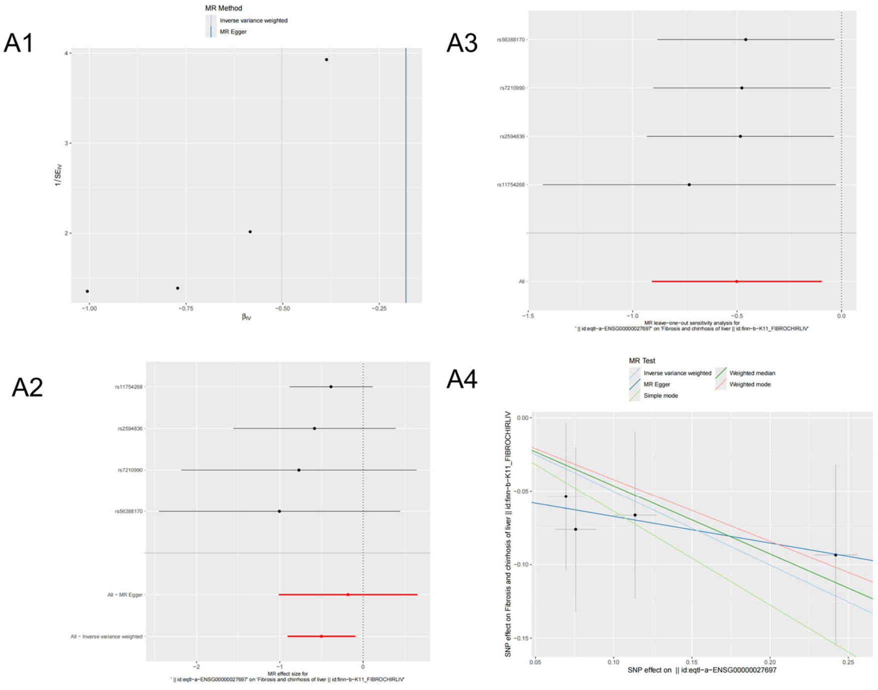

INFGR1

Figure S4. MR Graphical results of Liver Fibrosis and cirrhosis(finn-b-K11_FIBROCHIRLIV) .A1: funnel plots ; A2: OR scatter plots; A3:Leave-One-Out plots; A4: OR forest plots.OR=Odds

Ratio

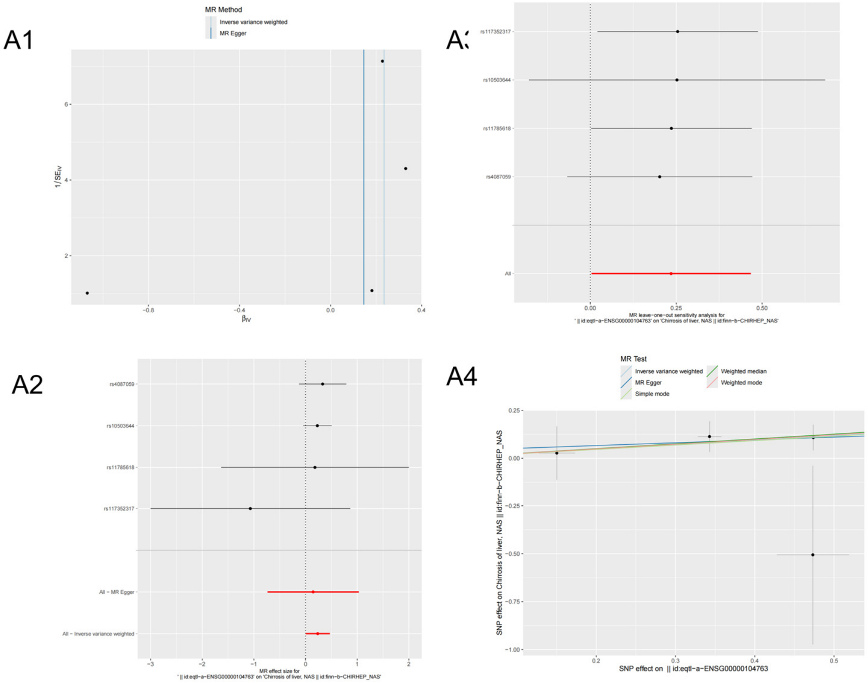

ASAH1

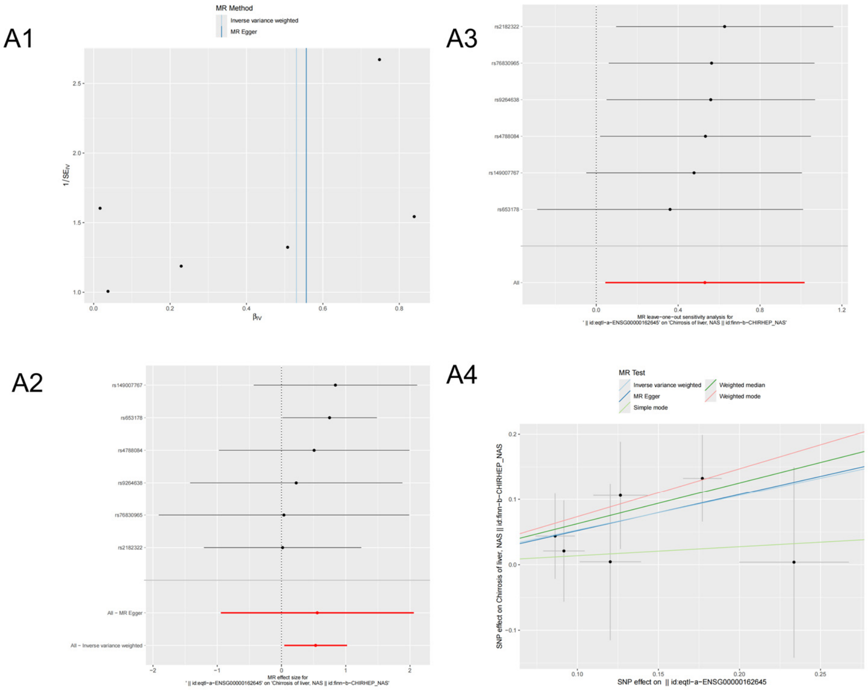

GBP2

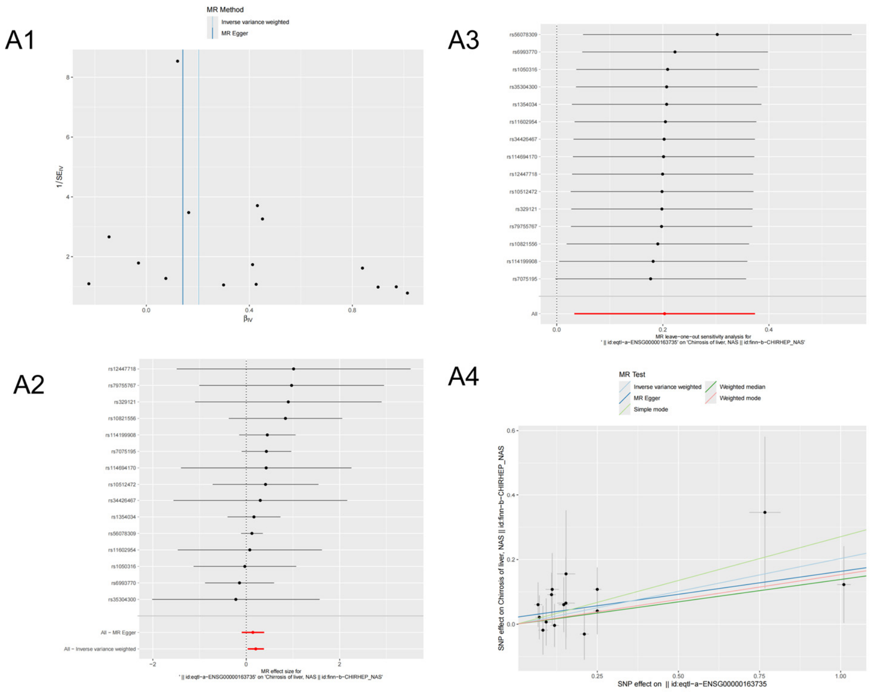
A1
A2
A3
A4
MR Method
Inverse variance weighted
MR Egger
MR Test
Weighted median
Weighted mode
Simple mode
SNP effect on || id:eqtl-a-ENSG00000163735

CXCL5

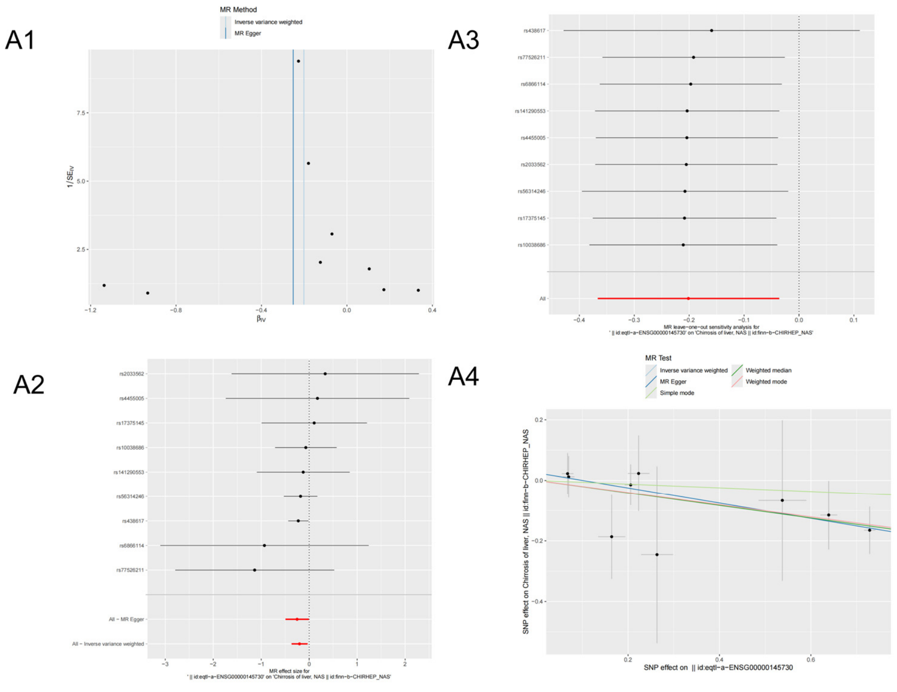
A1
A2
A3
A4
MR Method
Inverse variance weighted
MR Egger
MR Test
Weighted median
Weighted mode
Simple mode
SNP effect on || id:eqtl-a-ENSG00000145730

PAM

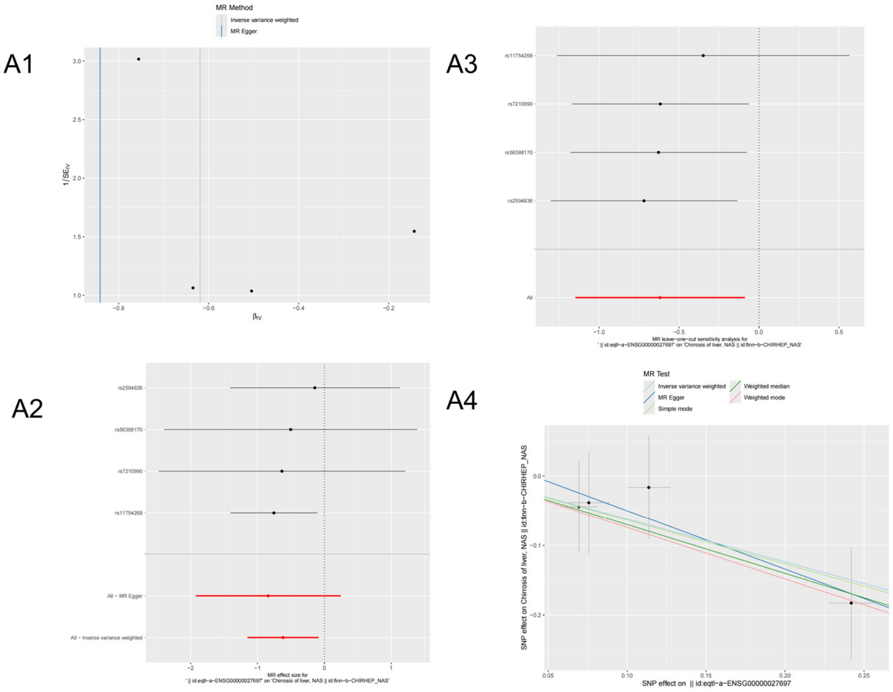

IFNGR1

Figure S5. MR Graphical results of cirrhosis of liver (finn-b-CHIRHEP_NAS) .A1: funnel plots ; A2: OR scatter plots; A3:Leave-One-Out plots; A4: OR forest plots.OR=Odds Ratio

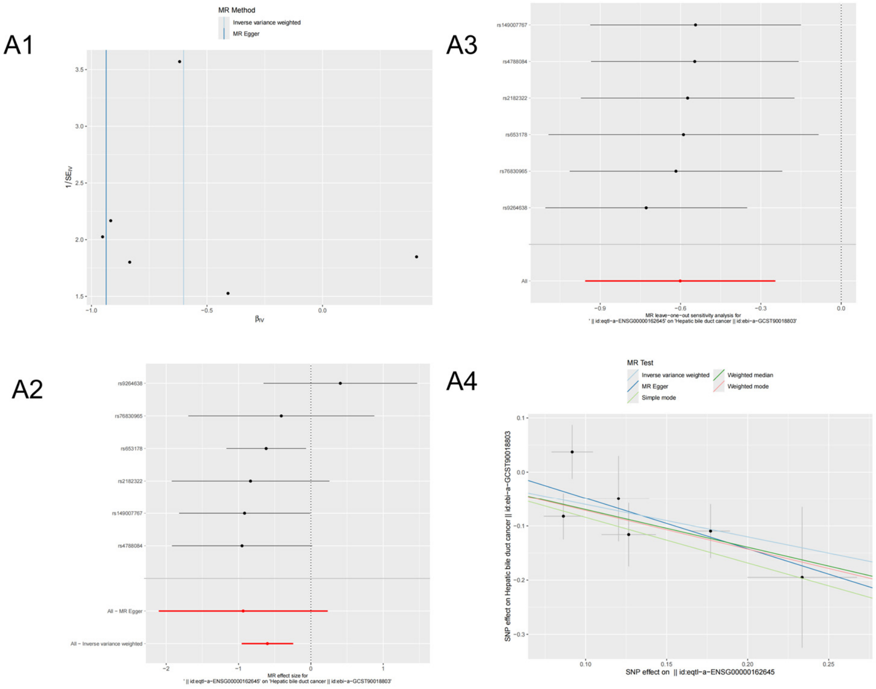

GBP2

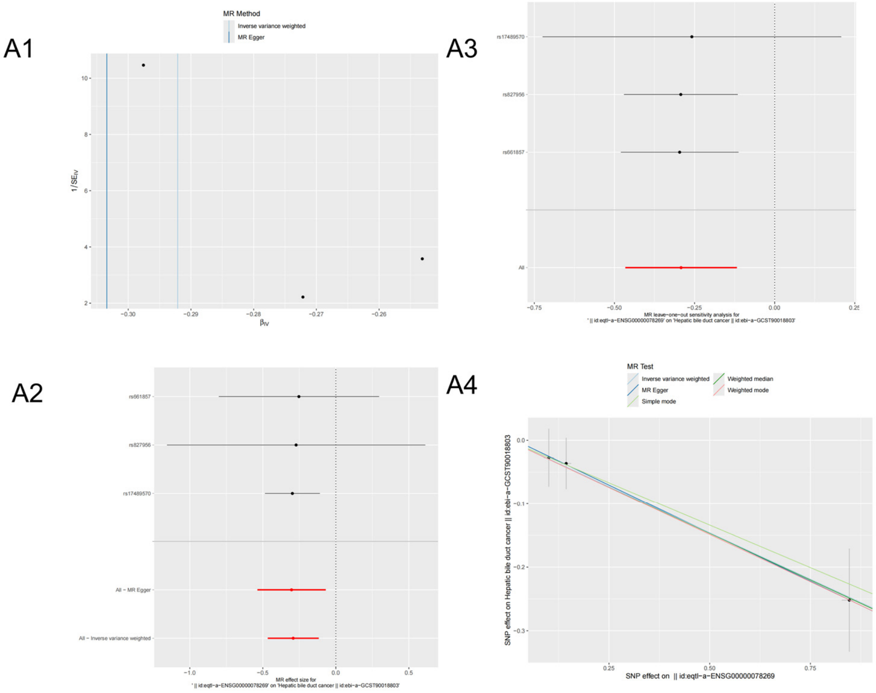

SYNJ2

Figure S6. MR Graphical results of Hepatic Bile Duct Cancer (ebi-a-GCST90018803) .A1: funnel plots ; A2: OR scatter plots; A3:Leave-One-Out plots; A4: OR forest plots.OR=Odds Ratio

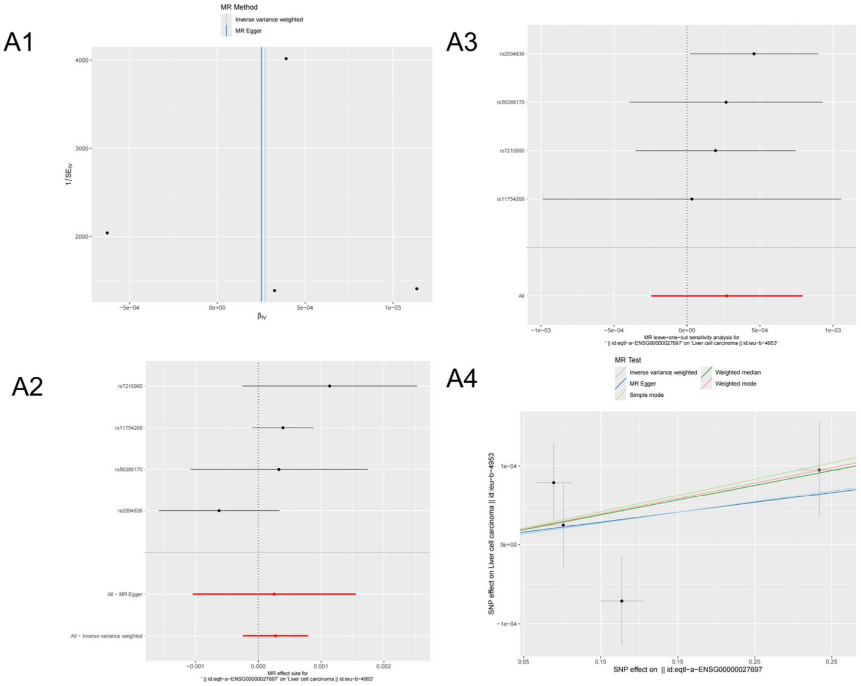
A1
MR Method
Inverse variance weighted
MR Egger
A3
A2
A4
MR Test
Inverse variance weighted
Weighted median
MR Egger
Weighted mode
Simple mode
SNP effect on Liver cell carcinoma || id:ieu-b-4953
SNP effect on || id:eqtl-a-ENSG00000027697

IFNGR1

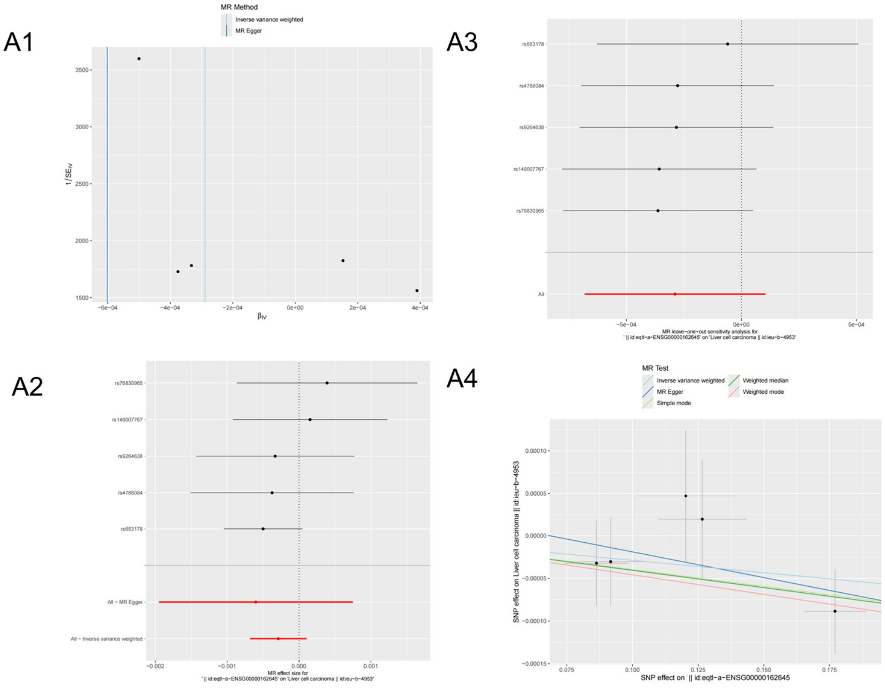
A1
MR Method
Inverse variance weighted
MR Egger
A3
A2
A4
MR Test
Inverse variance weighted
Weighted median
MR Egger
Weighted mode
Simple mode
SNP effect on Liver cell carcinoma || id:ieu-b-4953
SNP effect on || id:eqtl-a-ENSG00000162645

GBP2

Figure S7. MR Graphical results of Liver cell carcinoma (ieu-b-4953) .A1: funnel plots ; A2: OR scatter plots; A3:Leave-One-Out plots; A4: OR forest plots.OR=Odds Ratio

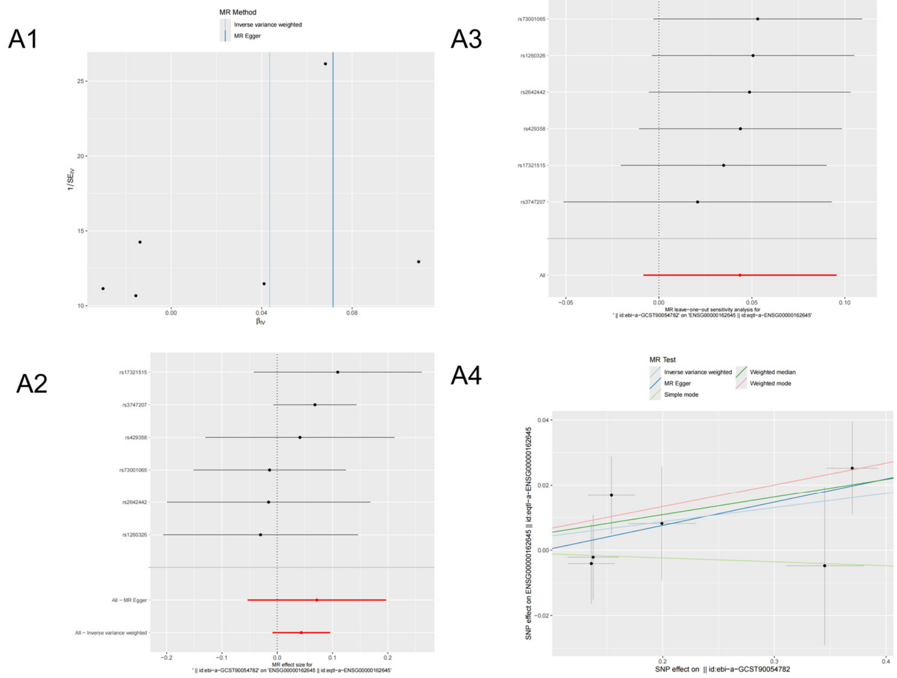

GBP2

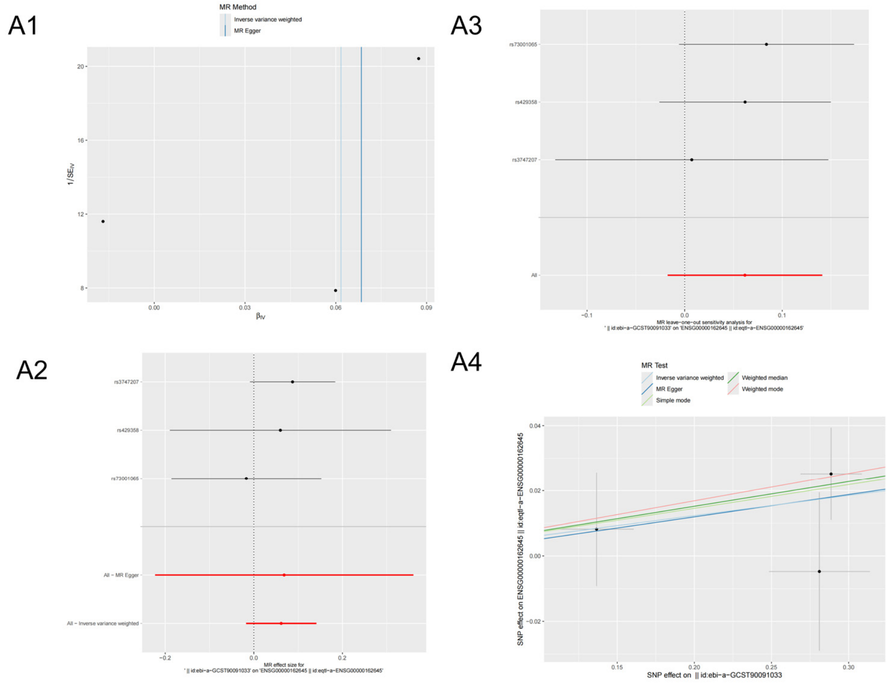

GBP2

Figure S8. Reverse MR of GBP2 on MASLD(ebi-a-GCST90054782) (UP)and iCCA(ebi-a-GCST90018803)(DOWN)

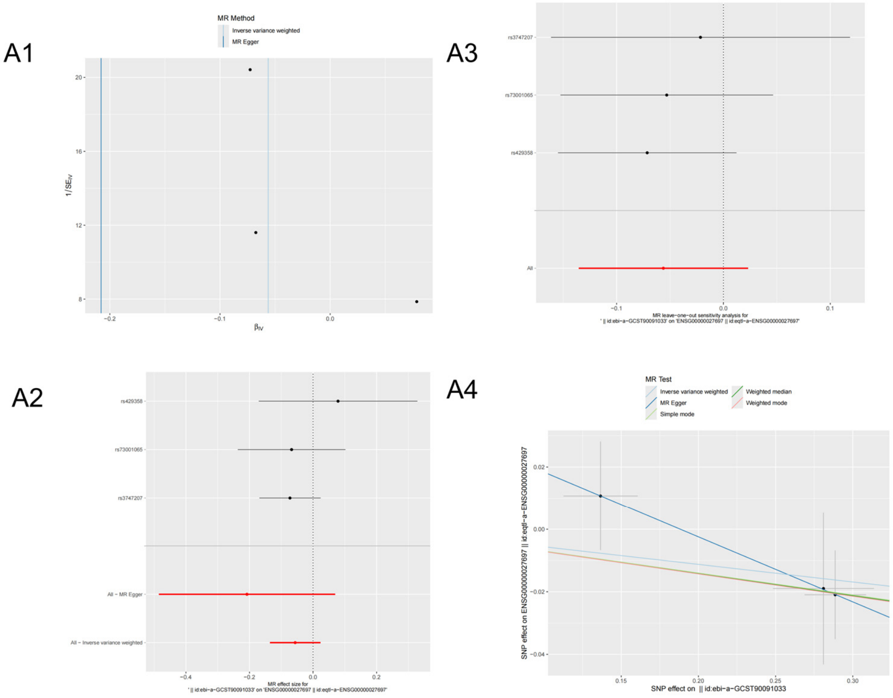

IFNGR1

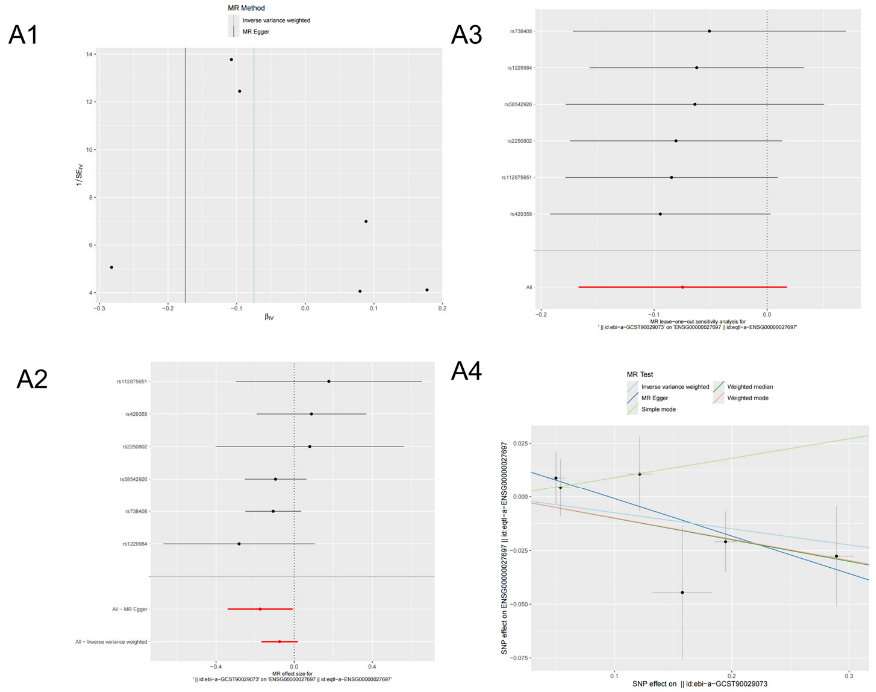

IFNGR1

Figure S9. Reverse MR of IFNGR1 on iCCA(ebi-a-GCST90018803) (UP)and liver fat(ebi-a-GCST90029073)(DOWN)

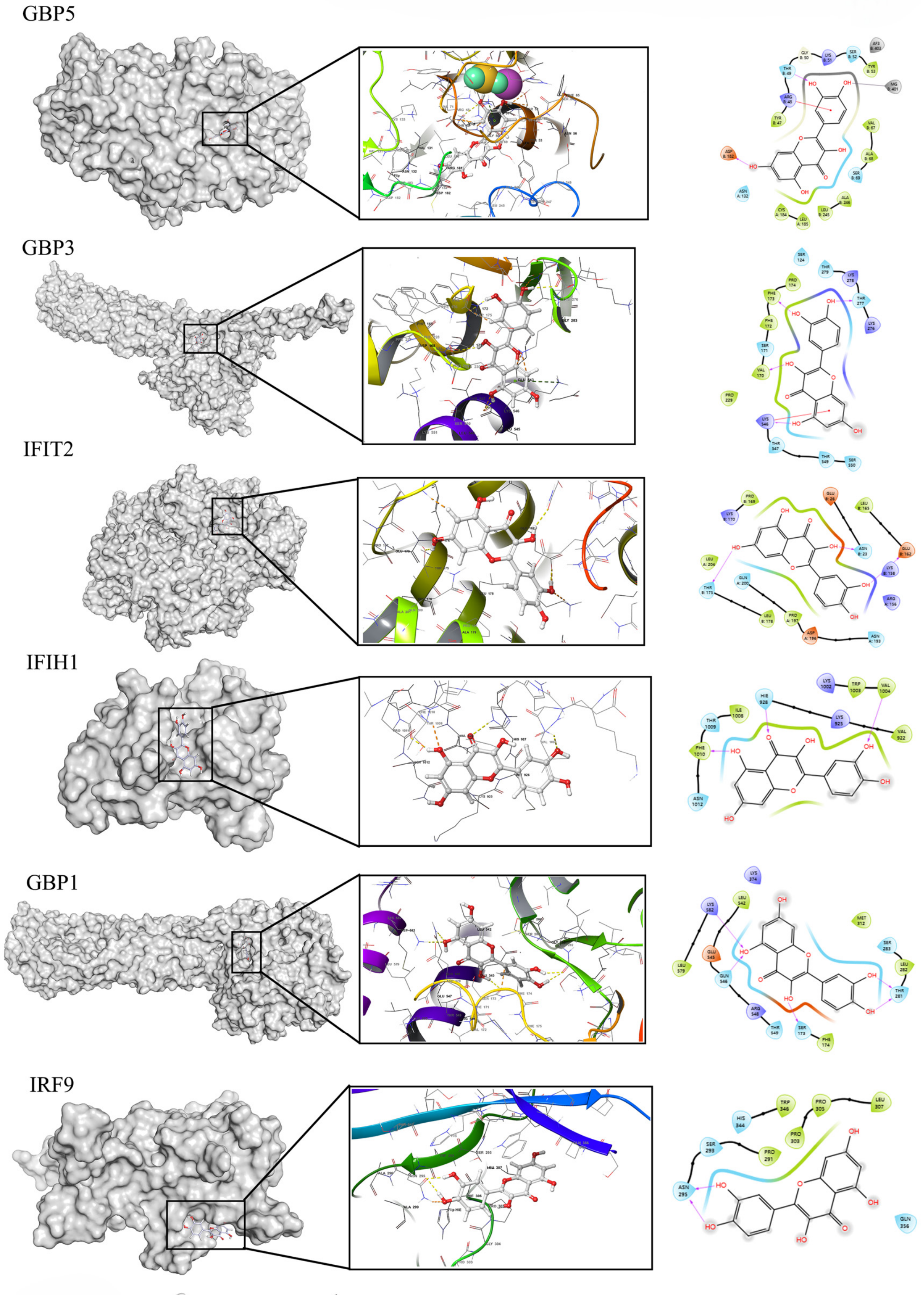
GBP5
GBP3
IFIT2
IFIH1
GBP1
IRF9

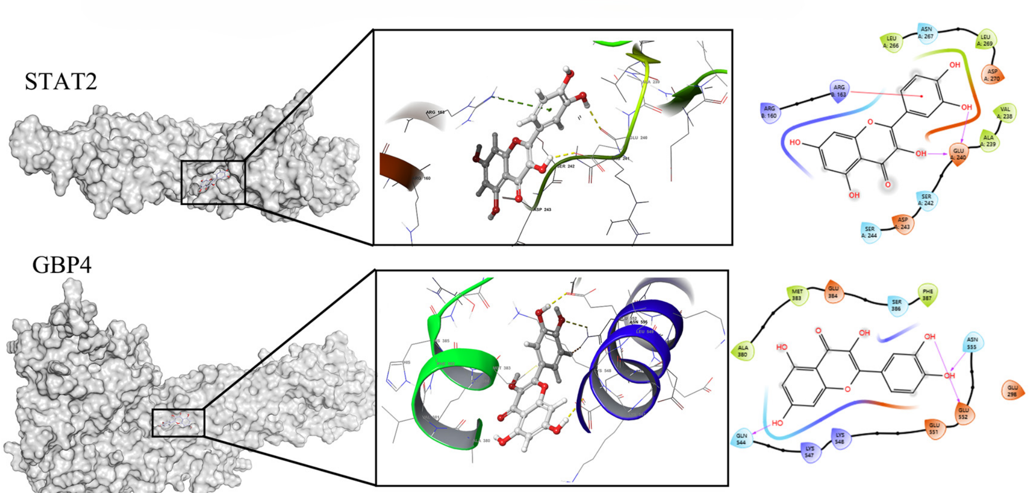

Figure S10. Docking results of quercetin with GBP2 related proteins. The docking score from top to bottle were GBP5:-7.523, GBP3:-6.777, IFIT2:-6.675, IFIH1:-6.427, GBP1:-6.269, IRF9:-6.179, STAT2:-5.209, GBP4:-5.172.

Table S3: cell-type-specific genes For GSE138709

Hepatocytes	Cholangiocytes	B cells	T cells	Dendritic cells	Endothelial cells	Macrophages	NK cells	Fibroblasts	Malignant cells
APOC3	FXYD2	MS4A1	CD2	CLEC9A	ENG	CD14	CD7	ACTA2	EPCAM
FABP1	TM4SF4	CD79A	CD3D	CD1C	VWF	LYZ	FGFBP2	COL1A2	KRT19
APOA1	ANXA4		CD3E	IFNGR1	GBP2	C1QB	KLRF1		KRT7

Table S4: cell-type-specific genes For GSE136103

Hepatocytes	Cholangiocytes	B cells	T cells	Dendritic cells	Endothelial cells	Macrophages	NK cells	Fibroblasts	Monocytes
APOC3	FXYD2	MS4A1	CD2	CLEC9A	ENG	CD14	CD7	ACTA2	FCN1
APOA1	TM4SF4	IGHA1	CD3D	CD1C	VWF	LYZ	FGFBP2	COL1A2	CSTA
Hepatocytes	ANXA4	MZB1	CD3E	IFNGR1	GBP2	C1QB	KLRF1		FCN2
ALB						MS4A7			
TF						CD163			
CYP									
